# Recent advances in nanomaterials and their mechanisms for infected wounds management

**DOI:** 10.1016/j.mtbio.2025.101553

**Published:** 2025-02-04

**Authors:** Jianping Zhu, Fan Xia, Shuaifei Wang, Yan Guan, Fuqiang Hu, Fangying Yu

**Affiliations:** a Department of Pharmacy, Sir Run Run Shaw Hospital, School of Medicine, Zhejiang University, Hangzhou, 310016, China; b College of Pharmaceutical Sciences, Zhejiang University, Hangzhou, 310058, China; c Department of Ultrasound in Medicine, Sir Run Run Shaw Hospital, School of Medicine, Zhejiang University, Hangzhou, 310016, China

**Keywords:** Nanomaterials, Antibacterial mechanism, Infected wounds, Drug delivery

## Abstract

Wounds infected by bacteria pose a considerable challenge in the field of healthcare, particularly with the increasing prevalence of antibiotic-resistant pathogens. Traditional antibiotics often fail to achieve effective results due to limited penetration, resistance development, and inadequate local concentration at wound sites. These limitations necessitate the exploration of alternative strategies that can overcome the drawbacks of conventional therapies. Nanomaterials have emerged as a promising solution for tackling bacterial infections and facilitating wound healing, thanks to their distinct physicochemical characteristics and multifunctional capabilities. This review highlights the latest developments in nanomaterials that demonstrated enhanced antibacterial efficacy and improved wound healing outcomes. The antibacterial mechanisms of nanomaterials are varied, including ion release, chemodynamic therapy, photothermal/photodynamic therapy, electrostatic interactions, and delivery of antibacterial drugs, which not only combat bacterial infections but also address the challenges posed by biofilms and antibiotic resistance. Furthermore, these nanomaterials create an optimal environment for tissue regeneration, promoting faster wound closure. By leveraging the unique attributes of nanomaterials, there is a significant opportunity to revolutionize the management of infected wounds and markedly improve patient outcomes.

## Graphical abstract

**Figure:**
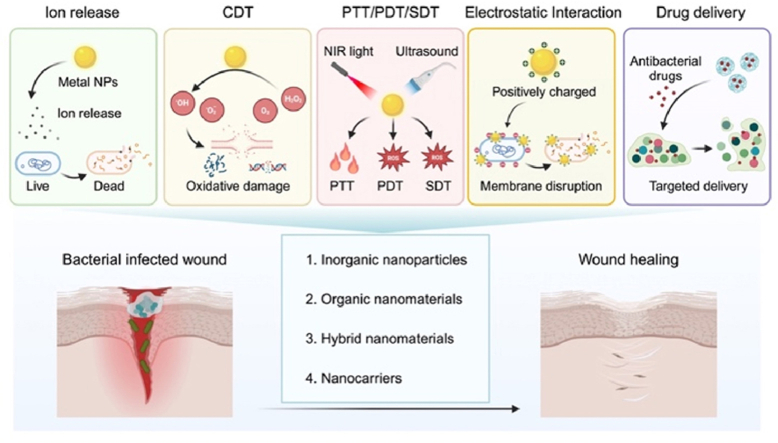

## 1 Introduction

Skin, as the largest organ of human body, possesses complex and multifunctional structures that play essential roles, including protection against physical, chemical, and biological threats, sensation, thermoregulation, and maintaining hydration balance [1,2]. As the primary barrier between internal and external environments, skin shields internal organs from external pathogens and environmental factors. However, when this protective barrier is compromised due to injury, surgery, or chronic conditions, it creates an entry point for pathogens, such as *Staphylococcus aureus* (*S. aureus*), methicillin-resistant *S. aureus* (MRSA), and *Pseudomonas aeruginosa* (*P. aeruginosa*) [3]. These pathogens can lead to wound infections, resulting in complications such as non-healing wounds, sepsis, and even death. Particularly, in individuals with underlying conditions such as diabetes, wound infections can easily become chronic, as diabetes can impair wound healing through impaired vascularization, neuropathy, and immune dysfunction [4]. Chronic wounds are often characterized by sustained inflammation, biofilm formation, and tissue necrosis, ultimately hindering wound closure and tissue regeneration [[5], [6], [7]]. If left untreated, chronic infections of wounds can result in serious complications, including heightened risk of amputation, systemic infections, extended hospital stays, and a markedly diminished quality of life.

Infected wounds create a hostile microenvironment that complicates treatment. The key features of this microenvironment mainly include elevated levels of reactive oxygen species (ROS), hypoxia, abnormal pH, and biofilm formation [8]. Initially, the immune response triggers acute inflammatory response, marked by the release of pro-inflammatory cytokines and recruitment of immune cells such as neutrophils and macrophages, which, although essential for pathogen clearance, can become dysregulated, leading to the generation of excessive ROS and exacerbating tissue damage. Persistent infections promote bacteria aggregation and biofilm formation, where extracellular polymeric substances (EPS) reduce bacterial metabolism and enhance resistance to conventional therapies [[9], [10], [11], [12]], making infections difficult to eradicate [13]. The metabolic activities of bacteria, such as fermentation and anaerobic respiration, generate acidic byproducts like lactic acid, leading to a local decrease in pH. Conversely, specific conditions such as diabetic wounds may exhibit a more alkaline environment due to altered metabolic pathways or imbalanced inflammation [[14], [15], [16]]. Additionally, impaired vascularization and bacterial metabolic activity deplete oxygen, resulting in hypoxia, which limits critical repair processes like angiogenesis and tissue regeneration. Together, these factors create a highly complex microenvironment, posing significant challenges for effective therapeutic interventions.

Traditional wound care strategies for managing infections rely heavily on systemic antibiotics [17] and conventional dressings [18]. However, antibiotic resistance, biofilm formation, and systemic toxicities often limit their efficacy. The mechanisms underlying antibiotic resistance are multifaceted [19], including: 1) Bacteria produce enzymes like β-lactamases that degrade antibiotics; 2) Mutations of the target sites of antibiotics, rendering them ineffective; 3) Altered membrane permeability of bacteria that reduces antibiotic uptake; 4) Efflux pumps actively expel antibiotics, lowering intracellular drug concentrations; 5) The protective microenvironment provides a protective niche for bacteria, and allows bacteria to evade immune responses and reduces their antibiotic sensitivity [13]. Additionally, systemic use of antibiotics can lead to off-target effects and contribute to resistance development due to their nonspecific action and prolonged use [20]. Furthermore, successful treatment of wound infections requires not only effective infection control but also the promotion of wound healing to restore skin integrity and function [21]. Consequently, a pressing requirement exists for innovative treatment approaches capable of efficiently tackling wounds infected by bacteria.

**Fig. 1:**
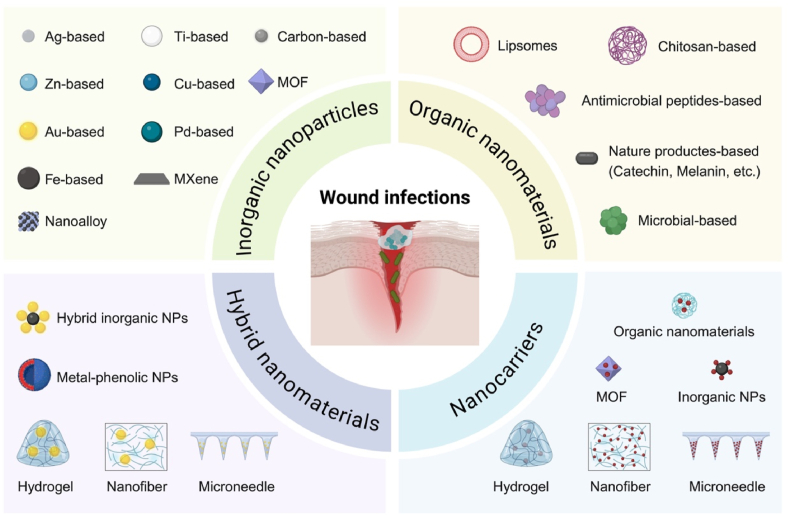
Schematic illustration of the types of nanomaterials used for managing infected wounds, including inorganic nanoparticles, organic nanomaterials, hybrid nanomaterials, and various nanocarriers. Nanomaterials exhibit unique physicochemical properties, such as inherent catalytic activities, high surface area-to-volume ratios, and tunable surface functionalities, which enables them eradicate pathogens, disrupt bacterial biofilms, and/or achieve controlled drug delivery and microenvironment modulation. This figure is created with BioRender.com.

In recent years, nanomaterials have emerged as promising candidates for robust antibacterial therapies and enhanced antibiotic delivery (Fig. 1) [19,[22], [23], [24]]. These include inorganic nanoparticles (NPs, e.g., silver (Ag), gold (Au), copper (Cu), titanium (Ti), iron (Fe), carbon dots (CDs), and metal-organic frameworks (MOFs)), organic nanomaterials (e.g., liposomes, chitosan-based NPs, peptides-based nanomaterials, nature products-derived nanomaterials, microbial-based nanomaterials), hybrid nanomaterials that combine the benefits of multiple components into a single multifunctional platform, and nanocarriers. Nanomaterials exhibit unique properties that make them highly effective against bacterial infections, particularly in addressing the challenges posed by infected wounds [[23], [24], [25]]. High surface area-to-volume ratios enhance interactions with bacterial membranes, leading to increased antibacterial activity through mechanisms like ion release and ROS generation. Tunable surface functionalities enable the development of multifunctional platforms that can adapt to wound microenvironments characterized by acidity, hypoxia, or inflammation. Furthermore, certain nanomaterials possess intrinsic catalytic, photothermal, or sonodynamic properties, allowing them to kill bacteria by disrupting membranes, generating localized heat, or producing ROS [7,26]. Nanocarriers offer controlled and sustained drug release, maintaining therapeutic concentrations over prolonged periods, reducing systemic toxicity, and enhancing penetration into biofilms [27,28]. Together, these properties position nanomaterials as versatile and powerful tools for managing bacterial infections.

**Fig. 2:**
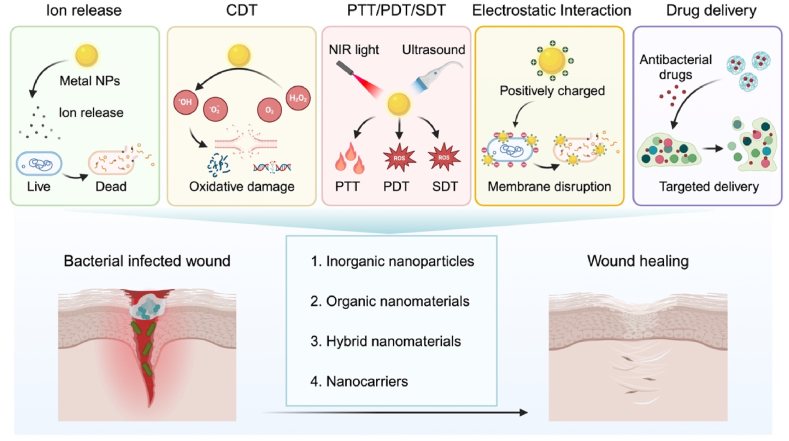
Schematic illustration of the antibacterial mechanisms of nanomaterials and their applications in the management of bacterial infected-wounds. Non-antibiotic antibacterial strategies include ion release, which damages bacterial membranes and inhibits essential cellular functions; CDT, which generates ROS to induce oxidative stress and bacterial death; PTT, PDT, and SDT, which utilize external stimulations to produce localized heat or ROS for targeted bacterial eradication; electrostatic interactions, where positively charged nanomaterials bind to negatively charged bacterial membranes, leading to structural disruption, increased membrane permeability, and eventual bacterial lysis. Antibiotic drug delivery systems enhance drug penetration into biofilms and enables sustained release for prolonged antibacterial effects. This figure is created with BioRender.com.

Unlike conventional antibiotics, which primarily interfere with bacterial cell walls, protein synthesis, or DNA replication, nanomaterials employ diverse and innovative antibacterial mechanisms that address the limitations of conventional therapies. These mechanisms can be broadly categorized into non-antibiotic antibacterial systems and antibiotic drug delivery systems (Fig. 2).

The mechanisms of non-antibiotic strategies mainly include ion release, chemodynamic therapy (CDT), photothermal, photodynamic, and sonodynamic therapies (PTT, PDT, SDT), and electrostatic interactions. 1) Ion release. Certain inorganic NPs can release metal ions that engage with bacterial cell membranes, resulting in structural damage, increased membrane permeability, and eventually cell lysis [29]. Unlike antibiotics that target specific molecular pathways and are prone to resistance development, ion release is a broad-spectrum mechanism effective against bacteria. For instance, AgNPs can emit Ag ions, which are particularly potent because of their capacity to attach to sulfur-containing proteins and phosphorus-rich DNA without requiring specific binding sites, bypassing traditional resistance mechanisms like target mutations [[30], [31], [32]]. 2) CDT. Nanomaterials such as Fe-based NPz, AgNPs [[30], [31], [32]], ZnO NPs [[33], [34], [35]], can catalyze the generation of ROS through Fenton or Fenton-like reactions to induce oxidative harm to bacterial cell, culminating in cellular dysfunction and mortality. As the effects of CDT are non-specific, it is less susceptible to resistance, and has been proved especially effective against a wide variety of bacteria. 3) PTT, PDT, and SDT. Certain nanomaterials, such as gold (Au) [36,37], copper (Cu) [[38], [39], [40]], and titanium dioxide (TiO_2_) NPs [41,42], possess photothermal, photodynamic, and/or sonodynamic properties. When subjected to NIR laser irradiation or ultrasound, these NPs can produce localized heat (PTT) or ROS (PDT or SDT), leading to bacterial cell destruction [26]. Unlike antibiotics, the non-specific nature of heat or ROS generation ensures that even antibiotic-resistant strains are vulnerable. 4) Electrostatic interactions. Positively charged nanomaterials, such as chitosan-based NPs and cationic CDs [[43], [44], [45], [46], [47]], interact strongly with the negatively charged bacterial cell walls, leading to membrane disruption, increased permeability, and bacterial death. This mechanism is distinct from antibiotics that require receptor-mediated entry or specific enzymatic interactions, thus bypassing bacterial resistance strategies like efflux pumps and enzymatic degradation.

Antibiotic drug delivery systems focus on enhancing the efficacy of traditional antibiotics through targeted and sustained release. Specialized nanocarriers can be designed to transport antibiotics directly to the infection site, thus enhancing local drug concentrations and minimizing off-target effects [27,28]. Unlike systemic antibiotic delivery, which often fails to penetrate biofilms or sustain effective concentrations, nanocarriers improve antibiotic infiltration within biofilms and enable controlled release. This sustained delivery reduces the frequency of administration and lowers the risk of resistance development by maintaining therapeutic levels over time.

In addition to their potent antibacterial effects, nanomaterials can be strategically designed to support tissue repair and promote the healing of infected wounds [48]. For example, nanomaterials, such as ceria nanozymes, mitigate chronic inflammation by scavenging excess ROS and promoting macrophage polarization from the pro-inflammatory M1 phenotype to the pro-healing M2 phenotype. Additionally, nanomaterials, such as Cu- and Zn-based NPs, stimulate angiogenesis by releasing bioactive ions to activate vascular endothelial growth factor (VEGF), while oxygen-generating nanomaterials alleviate hypoxia, further promoting neovascularization. Moreover, nanomaterials integrated into scaffolding materials, such as hydrogels or nanofibers, provides structural support for cell adhesion, migration, and proliferation, leading to organized tissue regeneration [[49], [50], [51], [52], [53], [54], [55], [56]]. Besides, multifunctional nanoplatforms combining antibacterial and regenerative features can simultaneously eradicate bacterial infections and create a favorable microenvironment for tissue repair. These diverse and synergistic mechanisms position nanomaterials as innovative tools for overcoming the challenges associated with infected wounds and achieving more effective and tailored therapeutic outcomes.

This review focuses on recent advancements in nanomaterials developed over the past five years for treating bacteria-infected wounds. By providing a comprehensive overview of these cutting-edge technologies, including non-antibiotic antibacterial systems and antibiotic drug delivery systems, we aim to deepen the understanding of their role in addressing the challenges of infection management. Additionally, this review highlights the diverse antibacterial mechanisms of nanomaterials, their advantages over traditional treatments, and the transformative potential of nanotechnology in wound care. These innovative strategies offer enhanced antibacterial efficacy, effective biofilm disruption, and accelerated tissue regeneration, paving the way for improved clinical outcomes in managing infected wounds.

## 2 Nanomaterials for non-antibiotic antibacterial therapies in wound infections

The rising prevalence of antibiotic resistance has necessitated the development of innovative, non-antibiotic strategies for managing wound infections. Nanomaterials offer unique advantages by leveraging mechanisms such as ion release, CDT, PTT, PDT, SDT, and electrostatic interactions (Table 1). This section highlights the rational design and application of nanomaterials with non-antibiotic antibacterial properties, emphasizing their ability to inhibit microbial growth, and create an environment conducive to wound healing.

**Table 1:** Summary of nanomaterials for infected wound management.s

Nanosystems	Antibacterial component	Antibacterial spectrum	Antibacterial mechanism	In vivo applications	Ref
L-AgÅPs-gel	AgNPs	*S. aureus*, MRSA, and *E. coli*	Release of Ag^+^	MRSA-infected wounds	[60]
Ag-Bi@SiO_2_ NPs	Ag-Bi NPs	MRSA	Enhanced Ag^+^ release under PTT	MRSA-infected wounds	[138]
Ag-G4/hemin	AgNPs	*S. aureus,* MRSA*,* and *E. coli*	Enhanced Ag^+^ release by oxidation	MRSA-infected wounds	[61]
Ag-P&C NPs	AgNPs	MRSA, and *E. coli*	pH-triggered size increase in Ag-P&C NPs, release of Ag^+^, and CDT effect	MRSA-infected wounds	[63]
polyCu-MOF@AgNPs	AgNPs	*S. aureus*	Release of Ag^+^, and ROS generation	*S. aureus* infected wounds	[64]
Cu-PTA NPs	Metal phenolic network composed by Cu^2+^ and PTA	*S. aureus*, *E. coli*, *S. mutans*, MRSA, and *P. aeruginosa*	Noncovalent interactions between the hydroxyl group of PTA scaffold induce the disruption of the bacterial membrane, and Cu^2+^ mediated ROS generation	*S. aureus*- or *P. aeruginosa*- infected wounds	[66]
PDA@ZnO NPs	PDA, and ZnO NPs	*E. coli*, and *S. aureus*	Release Zn ions and form ROS	*S. aureus*-infected wounds	[67]
CS@Fe-N CDs	Fe/N-doped CDs	*E. coli*, and *S. aureus*	CDT	*S. aureus*-infected wounds	[71]
CSPA NPs	Pt-Au NPs	*E. coli*, and *S. aureus*	CDT	Mixed bacterial (*E. coli* and *S. aureus*)-infected wounds	[74]
CFNSs	Cu_1.5_Mn_1.5_O_4_ nanospheres	*E. coli*, and MRSA	CDT	MRSA-infected wounds	[75]
GC@Pd	PdNPs	*S. aureus*	CDT	*S. aureus* infected wounds	[76]
APCR	Co-Ru NPs	MRSA	CDT	MRSA-infected diabetic wounds	[77]
Ni-MOF	Ni-based MOF	*E. coli*, and MRSA	CDT	*S. aureus*-infected wounds	[81]
FMA NPs	Fe_3_O_4_@MOF@AuNPs	*E. coli*, and *S. aureus*	CDT	Mixed bacterial (*E. coli* and *S. aureus*)-infected wounds	[82]
CG-KCD	Kanamycin-derived CDs	*E. coli*, and *S. aureus*	PDT, and the intrinsic effects from the retained active structure of kanamycin	*S. aureus*-infected wounds	[89]
Janus liposozyme	2-((5′-(4-(diphe- nylamino) phenyl)-[2,2′-bithiophen]-5-yl)methylene)malononitrile (TDTM)	MRSA	PDT	*S. aureus-*infected diabetic wounds	[91]
BBR@SP gel	*Spirulina platensis* (SP) and berberine (BBR)	MRSA	PDT	MRSA*-*infected diabetic wounds	[95]
mPDA@DFO@CP-SNO	mPDA, chitosan-graft-third generation poly- (amidoamine) polymer with terminal S-nitrosothiol groups (CP-SNO)	*E. coli*, and *S. aureus*	PTT, and direct antibacterial effects of NO	*S. aureus*-infected wounds	[96]
MCS@PDA@GCS NPs	PDA	MRSA, and *E. coli*	PTT	MRSA-infected wounds	[97]
Fe-CDs	Fe-doped CDs	*S. aureus,* and *E. coli*.	PTT, and PTT enhanced CDT	*E. coli* or *S. aureus*-infected wounds	[98]
LAMC/CD-C@M@P	Ceria oxide-molybdenum disulfide NPs with a PDA layer (C@M@P)	*S. aureus*, and *E. coli*	PTT	*S. aureus*-infected diabetic wounds	[99]
CINP@ZP-F127	Melanin-based NPs	MRSA, and *E. coli*	PTT	MRSA or *E. coli-*infected wounds	[104]
AMPs	Melanin-based NPs	*E.coil*	PTT	Linear fracture wound	[105]
PHMB@Au NPs	AuNPs	*S. aureus*	PTT	*S. aureus*-infected wounds	[37]
CuO@AgO/ZnO NPs	CuO NPs, AgO NPs, and ZnO NPs	*S. aureus*, and *P. aeruginosa*	PTT, and intrinsic antimicrobial activity of ZnO NPs and AgO NPs	*S. aureus*-infected wounds	[108]
TA-Fe/Cu nanocapsules	TA-Fe/Cu	MRSA, and *E. coli*	PTT, and inherent antibacterial properties derived from TA	MRSA-infected diabetic wounds	[114]
FPSa@M hydrogel	Ti_3_C_2_T_X_ MXene	*E. coli*, *S. aureus*, and MRSA	PTT	MRSA-infected and burn wounds	[117]
Pec@PLL-MoS_2_	MoS_2_	*E. coli,* and *S. aureus*	PTT	Mixed bacterial (*E. coli,* and *S. aureus*)-infected wounds	[119]
HEA@Gel	*Haematococcus* (HEA) cells	*E. coli,* and *S. aureus*	PTT	*S. aureus-*infected diabetic wounds	[120]
BTO@MM_Sa_	BTO NPs	*S. aureus*, and *E. coli*	SDT	*S. aureus*-infected wounds	[123]
GQDs/TiO_2−x_	TiO_2−x_ microspheres	*E. coli*, *S. aureus*, and MRSA	SDT	MRSA-infected wounds	[124]
ZnLiPOI	Li-doped ZnO	*E. coli,* and *S. aureus*	SDT	*S. aureus*-infected wounds	[125]
CS@PLCL/DWJM@Cu nanofiber	Chitosan	*E. coli* and *S. aureus*	Inherent antimicrobial capabilities of chitosan	Diabetic wounds	[43]
CDs	Positively charged CDs	*S. aureus*	CDs with positive charge on the surface generate strong static electricity with the negatively charged bacterial film	*S. aureus*-infected wounds	[44]
HA-LM2R-MR dressing	RWRWRW peptide	MRSA*,* and *P. aeruginosa*	Cationic RWRWRW interact with negatively charged microbial membrane	MRSA*-*infected diabetic wounds	[45]
mWRWRWY NPs	WRWRWY peptide	*E. coli* and *S. aureus*	WRWRWY self-assembly into positively charged NPs to interact with negatively charged microbial membrane	*S. aureus-*infected wounds	[46]
PtCuTe nanosheets	PtCuTe nanosheets	*S. aureus*, and *E. coli*	Te-mediated membrane damage and flagellar movement inhibition	*S. aureus*-infected diabetic wounds	[132]
MB NPs	Nano-MgB_2_	*P. aeruginosa*	Generate boron dihydroxyl groups and metal cations to form stable borate ester bonds with LPS or PGN	*P. aeruginosa*-infected wounds	[133]
Cu^2+^-based am-MPN NPs	Metal phenolic network composed by EGCG, Cu^2+^, and DEDTC	*S. epidermidis*, MRSA, *E. coli*, and *P. aeruginosa*	Disruption of cell walls, generation of ROS, and formation of quinoproteins	MRSA-infected wounds	[134]
GA-Ag NPs	AgNPs	*S. aureus*, and *E. coli*	Release of Ag^+^, and PTT	*S. aureus*-infected wounds	[137]
Cu_2.8_O@silicene-BSA	Cu_2.8_O NPs	MRSA	CDT, and PTT	MRSA-infected wounds	[40]
DM/Cu^2+^-CuS NPs	Cu-based	*S. aureus,* and *E. coli*	CDT, and PTT	*S. aureus*-infected wounds	[139]
FeCP/ICG@CaO_2_	FeCP, ICG	MRSA*,* and *KREC*	CDT, and PTT	MRSA-infected skin tumor wounds	[140]
PFG/M MNs	PDA, iron oxide NPs	*S. aureus*, and *E. coli*	CDT, and PTT	*S. aureus*-infected wounds	[141]
PdNS	Pd nanosheets	*Ec. faecium*, *S. aureus*, *K. pneumoniae*, *A. baumannii*, *P. aeruginosa*, and *Eb. Hormaechei.*	CDT, PTT, PDT, and mechanically disruption of cell membranes,	*S. aureus-* or *P. aeruginosa-*infected wounds	[142]
P-bioHJ	Ti_3_C_2_ quantum dots, and FeS NPs	*E. coli*, and *S. aureus*	CDT, PTT, and PDT	*S. aureus*-infected wounds	[143]
CuS@TA-Fe NPs	CuS, and metal phenolic network composed by TA and Fe^3+^	*E. coli*, and *S. aureus*	CDT, PTT, and PDT	*S. aureus*-infected wounds	[144]
PDG@Au–NO/PBAM	AuNPs conjugated PDG NPs	MRSA, and tetracycline-resistant *E. coli*	PTT, and NO gas release	MRSA*-*infected wounds	[147]
PSS NPs	PB NPs	MRSA	PTT, and NO gas release	MRSA*-*infected diabetic wounds	[148]
SNO-CS@MoS_2_	MoS_2_ NSs	*S. aureus*, and *E. coli*	PTT, and NO gas release	*E. coli*-infected wounds	[149]
ICG&CO@G3KBPY	ICG	*E. coli*, *S. aureus*, and MRSA	PTT, and CO gas release	MRSA*-*infected wounds	[151]
MCC/CS NPs	Chitosan and mono-carboxyl corrole	*E. coli,* and MRSA	Inherent antimicrobial capabilities of chitosan, and PTT	MRSA*-*infected wounds	[47]
mTiO_2_@PDA NPs	PDA modified mesoporous TiO_2_ NPs	*S. aureus*, and *E. coli*	PDT, PTT, and SDT	*E. coli*-infected wounds	[42]

### 2.1 Designing nanomaterials with ion release property

**Fig. 3:**
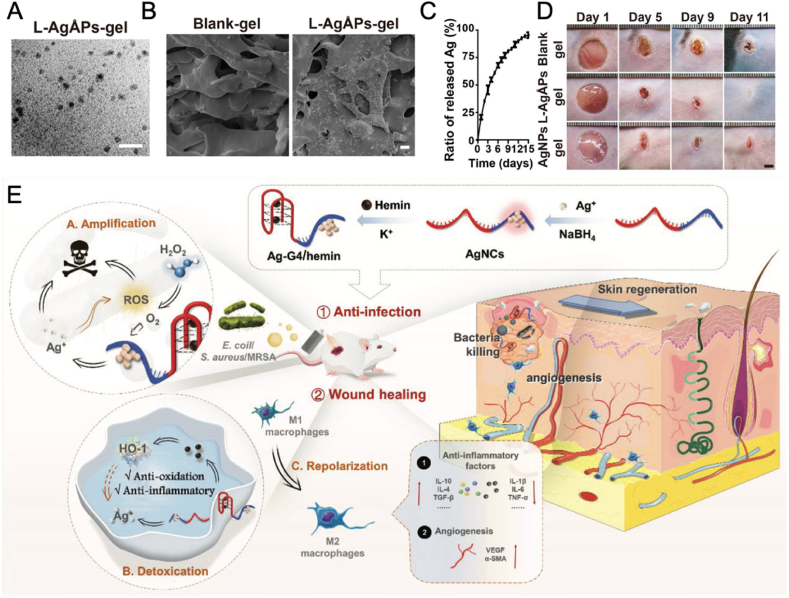
(A) TEM image of L-AgÅPs-gel (Scale bar, 20 nm). (B) Scanning electron microscopy (SEM) images of blank-gel and L-AgÅPs-gel (Scale bar, 10 μm). (C) The release curve of Ag^+^ ions from L-AgÅPs-gel. (D) Photographs of mice with diabetic wounds after treated with blank-gel, L-AgÅPs-gel, or AgNPs-gel (Scale bar, 2 mm) [60]. Copyright 2020, The American Association for the Advancement of Science. (E) Schematic illustration of the fabrication and the therapeutic mechanism against the infected wound of Ag-G4/hemin [61]. Copyright 2024, Wiley-VCH.

AgNPs have attracted considerable interest in the area of skin infection treatment because of their strong antimicrobial effects, broad-spectrum activity, and reduced chance of resistance formation compared to traditional antibiotics [[57], [58], [59]]. The main bactericidal mechanism is probably due to the discharge of Ag^+^ from AgNPs, which have a high tendency to bind with thiol groups found on cell membranes, interfering with cell division and eventually resulting in bacterial cell death. Recently, various strategies have been explored for enhancing the Ag^+^ and enhance their antibacterial capabilities, including decreasing particle size, utilizing external stimulations and internal stimulations, and increasing the loading efficiency of AgNPs. For example, Xie et al. decreased the particle size of AgNPs to Ångstrom-scale (65.9 ± 31.6 Å), which were then embedded in carbomer gel to obtaing L-AgÅPs-gel (Fig. 3A and B) [60]. The gel can achieve a sustained release of Ag^+^ ions in 15 days (with a released amount up to 94.38 ± 2.01 %) and demonstrate broad-spectrum antibacterial activity without significant toxicity to wound healing-related cells (Fig. 3C and D).

Excessive oxygen can oxidase AgNPs, thus enhancing the release of Ag^+^ ions. In G-quadruplex/hemin DNAzyme-functionalized silver nanoclusters (Ag-G4/hemin), the oxygen generated by G4/hemin enhances the oxidative release of Ag^+^ ions and strengthens the POD-like activity of AgNPs, resulting in excessive ROS production [61]. These mechanisms act synergistically to kill bacteria, including Gram-positive *S. aureus*, MRSA, and Gram-negative *Escherichia coli* (*E. coli*) (Fig. 3E). Additionally, the antioxidative properties of Ag-G4/hemin assist in shifting pro-inflammatory M1 macrophages toward the pro-healing M2 phenotype. This dual action enables Ag-G4/hemin to effectively manage MRSA-infected wounds by both eradicating bacteria and facilitating wound healing.

Typically, due to the accumulation of acidic byproducts within the EPS matrix, the biofilm microenvironment is weakly acidic [62]. Cheng et al. designed pH-sensitive and size-tunable AgNPs (Ag-P&C NPs) to enhance biofilm penetration, promote the ion release, and prolong retention at infection sites [63]. The Ag-P&C NPs were composed of Ag-peptide NPs (AgNPs engineered with NH_2_-Lys-Arg_4_-Gly-His_4_-Cys-CM) and Ag-CABT NPs (AgNPs engineered with 2-cyano-6-aminobenzothiazole). Under weakly acidic conditions, the protonated imidazole groups on histidine triggered surface charge transfer, promoting the penetration of Ag-P&C NPs into biofilms and enhancing the Ag^+^ ion release. Additionally, the click cycloaddition between cysteine on Ag-peptide NPs and the cyano group on Ag-CABT NPs facilitated aggregation, allowing for long-term retention of Ag-P&C NPs. As a result, Ag-P&C NPs showed potent antibacterial effects against MRSA and *E. coli*, and showed excellent performance in treating wounds infected with MRSA.

A direct but effective approach to enhance the ion release is increasing the loading efficiency of AgNPs. Polymer metal-organic frameworks (polyMOFs), with improved stability and precisely controlled structures, have gained attention for the delivery of therapeutic agents [64,65]. Guo et al. utilized a Cu-based polyMOF (polyCu-MOF) with an ultrathin nanosheet structure and a large surface area to facilitate the adsorption of a large amount of Ag ions, which were subsequently reduced to form polyCu-MOF@AgNPs [64]. The hybrid material exhibited reduced Cu^2+^ release and increased Ag ^+^ release compared to traditional Cu-MOF@AgNPs, thereby improving biocompatibility and lowering hemolysis risk. The polyCu-MOF@AgNPs effectively killed *E. coli* and *S. aureus* by disrupting cell integrity and bacterial metabolism in vitro. In mice with *S. aureus* infected wounds, polyCu-MOF@AgNPs not only enhanced wound healing through their synergistic antibacterial effects but also promoted skin regeneration and dense collagen deposition.

In addition to Ag^+^, other metal ions have also shown potential for antibacterial applications. Li et al. developed Cu-poly(tannic acid) NPs (Cu-PTA NPs) with high biocompatibility for the treatment of infected wounds [66]. The Cu-PTA NPs responded to elevated ROS levels in the wound microenvironment, releasing Cu^2^⁺ in a controlled manner. Subsequently, the NPs exhibited strong antibacterial effects by disrupting the bacterial membrane through noncovalent interactions between the hydroxyl group of PTA scaffold, and subsequent ∙OH generation mediated by Cu^2+^. Liao et al. developed collagen-based nanocomposite dressings (APZC) by incorporating polydopamine (PDA)-coated zinc oxide NPs (PDA@ZnO NPs) into a collagen matrix and crosslinking it with dialdehyde sodium alginate [67]. The PDA coating enhanced the interactions between NPs with the collagen matrix, ensuring their uniform distribution of NPs within the dressing. Moreover, the zinc ions released form ZnO NPs catalyzed the generation of abundant ROS, showing antibacterial action against *E. coli* and *S. aureus*. Rabbit model experiments demonstrated that these dressings significantly accelerated wound healing, reduced inflammation, and enhanced collagen deposition and angiogenesis.

Overall, therapeutic ions exert antibacterial effects through mechanisms including disrupting bacterial membranes, interfering with protein function, and generating ROS. Advances such as size optimization, stimuli-responsive systems, and hybrid nanostructures enhance targeted ion release, particularly against biofilms and drug-resistant bacteria. However, precise control over ion release to balance efficacy and biocompatibility remains a challenge. Future efforts should prioritize multifunctional platforms integrating ion release with regenerative and immune-modulating properties, while addressing scalability and safety for clinical use.

### 2.2 Designing nanomaterials for CDT

CDT has emerged as a promising antibacterial strategy, leveraging the unique ability of nanomaterials to catalyze the conversion of endogenous hydrogen peroxide (H₂O₂) into highly reactive ROS [68]. A growing number of nanomaterials have been found to possess intrinsic enzyme-like activities, such oxidase (OXD)-like, peroxidase (POD)-like, superoxidase (SOD)-like, and catalase (CAT)-like activities, thus can modulate the ROS levels in various diseases, as well as bacterial infections [69,70]. For example, Li et al. developed Fe/N-doped chitosan-chelated CDs (CS@Fe-N CDs) nanozyme, with exceptional stability, super POD-like activity, and robust antibacterial properties [71]. The CS@Fe-N CDs demonstrated high catalytic efficiency in converting H_2_O_2_ into ·OH, with a high V_max_/K_m_ ratio of 1.77 × 10^−6^/s, which significantly enhanced antibacterial activity against *S. aureus*. Subsequently, the nanozymes exhibited minimal cytotoxicity and accelerated wound healing in rats by at least 4 days, outperforming penicillin.

**Fig. 4:**
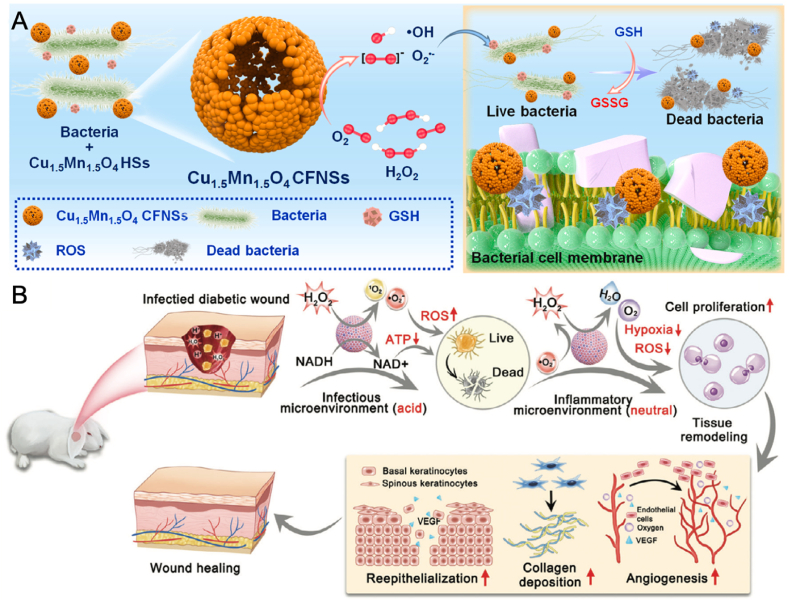
(A) Schematic illustration of the therapeutic mechanism of Cu_1.5_Mn_1.5_O_4_ CFNSs with multiple enzyme-like activities for wound infections [75]. Copyright 2022, Elsevier. (B) Illustration of the therapeutic mechanism of APCR for diabetic wound [77]. Copyright 2024, Wiley-VCH.

To enhance the enzyme-like activities of nanozymes, nanoalloys has emerged as innovative catalysts that overcome the limitations of single metal centers by utilizing the cooperative impact of multiple metals [72,73]. For example, Wen et al. fabricated chitosan-stabilized Pt-Au (CSPA) NPs with multienzyme-like activities to combat mixed bacterial infections in wound healing [74]. The CSPA exhibited strong OXD-, POD-, and NADH dehydrogenase-like activities, thus can generate excessive ROS to damage bacterial cells and disrupt their energy metabolism. Similarly, to enhance the catalytic activity and GSH depletion properties of Cu-based NPs, Wu et al. developed bimetallic oxide Cu_1.5_Mn_1.5_O_4_ cage-like frame nanospheres (CFNSs) with superior enzyme-mimic activities, offering a novel strategy for bacterial-infected wound therapy (Fig. 4A) [75]. The CFNSs, with their exposed active edge sites, exhibited exceptional triple enzyme-like activities, including OXD-, POD-, and glutathione peroxidase (GPx)-mimic activities, which promoted the generation of ROS. By inducing oxidative stress, CFNSs effectively killed bacteria and damage bacteria cells. In mouse model with MRSA infected-wounds, CFNSs were highly effective in disinfecting wounds and promoting healing, without causing detectable toxicity or adverse side effects.

Controlling the enzyme-like activities of nanozymes in different microenvironment to make them generating ROS in the antibacterial stage while eliminating ROS in the wound healing stage has shown great promise in the infected wound management. For example, Chen et al. coordinated gallic acid-modified chitosan (GC) with Pd NPs (GC@Pd) for treating biofilm-associated diabetic chronic wounds [76]. Under acidic biofilm conditions, GC@Pd exhibited OXD-like activity, generating ROS that effectively dismantle biofilms. As the wound environment shifts to alkaline during the healing process, GC@Pd transitions to CAT-like activity, scavenging excessive ROS and mitigating inflammation. In addition, the GC component can selectively capture pro-inflammatory cytokines through Michael addition reactions, further contributing to inflammation resolution. Both in vitro and in vivo studies confirmed that the GC@Pd was able to synergistically eliminate bacterial infections, reduce inflammation, regulate immune responses, and promote wound healing. Likewise, Gao et al. developed self-adaptive artificial peroxisomes embedded with cobalt-ruthenium (Co-Ru) centers (APCR), which performed dual functions depending on the microenvironment: producing ROS for antibacterial activity in acidic conditions and scavenging ROS to promote tissue regeneration and reduce inflammation in neutral conditions (Fig. 4B) [77]. The Co-Ru centers catalyzed the conversion of H_2_O_2_ into ROS, effectively destroying bacterial membranes and metabolic pathways, providing potent antimicrobial activity against drug-resistant bacteria like MRSA. After bacterial elimination, the APCR transitioned to ROS scavenging, alleviating oxidative stress and creating a favorable environment for wound healing.

MOFs have also emerged as versatile platforms for CDT and antibacterial applications [78]. MOFs can be engineered to fulfill various roles by carefully choosing metastable coordination bonds. By rational design, MOFs can be engineered to catalyze ROS generation, release antibacterial ions, or enhance the dispersion and catalytic activity of loaded nanoparticles [79,80]. Recently, Chen et al. fabricated a nickel-based MOF (Ni-MOF) that exhibits dual enzymatic activity contingent on the pH environment to promote infected wounds healing [81]. This Ni-MOF demonstrated POD-like activity in an acidic environment, generating hydroxyl radicals that effectively kill bacteria. Following bacteria eradication, the microenvironment of the wound gradually shifted towards a neutral state, in which Ni-MOF exhibited SOD-like activity, scavenging excess ROS and promoting the transition of macrophages to the anti-inflammatory M2 phenotype. As a result, this Ni-MOF can actively participate in different stages of the healing process, from bacterial disinfection to modulation of the inflammatory response. Additionally, MOFs with high porosity and accessible active sites can also serve as stabilizing and functionalizing agents, enhancing the dispersion and catalytic activity of the loaded NPs. For example, Liu et al. developed Fe_3_O_4_@MOF@AuNPs (FMA NPs), synthesized through the in-situ growth of ultra-small AuNPs on MOF-stabilized iron oxide NPs, for antibacterial wound healing without the use of antibiotics [82]. The inclusion of ultra-small AuNPs within the MOF layer improved the POD-like activity through a cascade reaction between Au and Fe_3_O_4_ NPs, enabling FMA NPs to produce ·OH from H_2_O_2_ at low concentrations (0.97 μM). In vitro and in vivo experiments revealed that the FMA NPs had excellent biocompatibility, and favorable antibacterial activity against both *E. coli* and *S. aureus*.

CDT employs nanomaterials with catalytic activities to generate ROS, providing effective bacterial eradication and biofilm disruption. Nanomaterials, including various inorganic NPs, bimetallic alloys, and MOFs demonstrate exceptional catalytic efficiency, showing great potential in the wound infections management. Moreover, various efforts have been applied for the synergistically improve ROS levels to enable antibacterial effects during infection while facilitating tissue regeneration during wound healing. Despite these advancements, precise control over ROS levels to avoid host tissue damage, along with improving biocompatibility and stability, remains a challenge. Therefore, addressing biocompatibility and clinical validation while maintaining their catalytic activities is necessary in the future research on CDT nanoplatforms.

### 2.3 Designing nanomaterials for PDT

**Fig. 5:**
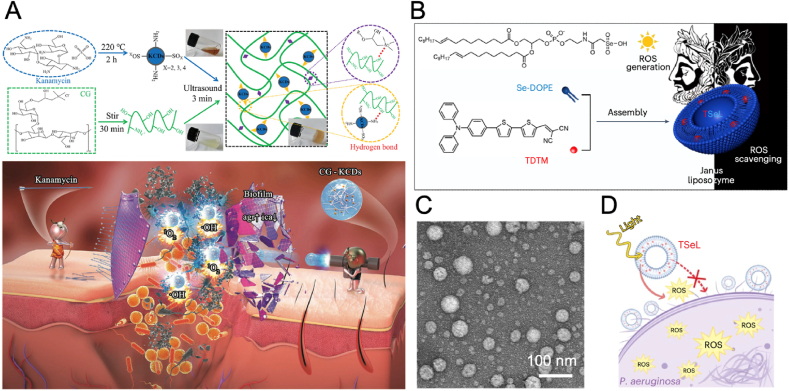
(A) Schematic diagram of the synthesis of CG-KCDs hydrogels and their mechanism for treating biofilm-associated wounds [89]. Copyright 2023, Wiley-VCH. (B) Schematic illustration of the preparation of Janus liposozyme. (C) Characterization of Janus liposozyme. (D) Illustration of the antibacterial mechanism of Janus liposozyme [91]. Copyright 2024, Springer Nature.

PDT offers a unique approach to combating bacterial infections by utilizing photochemical reactions to disrupt bacterial activity, addressing the complex challenges of infected wound management [83,84]. CDs are valued for their ease of synthesis, ultra-small size, excellent optical properties, low toxicity, and high biocompatibility, showing great potential for antibacterial applications [[85], [86], [87], [88]]. Wang et al. synthesized kanamycin-derived CDs (KCDs) with photodynamic and antibiotic activities, and were encapsulated within a cationic guar gum (CG) hydrogel matrix (CG-KCD) for treating biofilm-associated wounds (Fig. 5A) [89]. The KCDs not only exhibited photodynamic therapeutic efficiency but also retained the active structure of kanamycin while forming additional surface-modified components that enhanced antibacterial action, thus effectively disrupting bacterial biofilms. Under laser irradiation, the hydrogel caused significant biofilm disruption through ROS generation, reducing bacterial viability by damaging cell walls and membranes. In a mouse wound infection model, the hydrogel accelerated wound healing, reduced inflammation, and promoted tissue regeneration compared to controls. Moreover, the hydrogel-treated wounds also displayed better collagen deposition and re-epithelialization, indicating improved tissue remodeling.

Liposomes have recently attracted considerable interest for antibacterial therapies because of their superior biocompatibility and high efficiency in drug encapsulation [90]. Wei et al. developed an innovative Janus liposozyme composed by seleno-phospholipid and photosensitizers, which enabled the Janus liposozyme to mimic glutathione peroxidase activity for scavenging excess ROS and produce ROS upon light activation for antibacterial action (Fig. 5B–D) [91]. Subsequently, the Janus liposozyme not only targets bacterial pathogens like MRSA with photoactivated ROS production but also modulates the oxidative and immune status of wounds, promoting tissue healing. Both in vitro and in vivo studies demonstrated that the Janus liposozyme promoted macrophage polarization from M1 to M2 and facilitated tissue repair, with single-cell RNA sequencing revealing that this macrophage transition was mediated by IL-17-producing γδ T cells. In mouse and mini-pig models with infected diabetic wounds, the liposozyme significantly accelerated wound closure, enhanced collagen deposition, and stimulated angiogenesis.

Microorganisms-derived nanomaterials offer distinct advantages for antibacterial therapy due to their versatility and programmability [[92], [93], [94]]. Hu et al. integrated berberine (BBR), a quorum sensing (QS) inhibitor and antibacterial agent, with *Spirulina platensis* (SP), a natural oxygen generator and photosensitizer, into hydrogel to obtain a microalgae-based bioactive hydrogel (BBR@SP gel) for treating infected diabetic wounds [95]. Upon laser irradiation, the gel can simultaneously disrupt bacterial QS, reduce biofilm formation, and exert photodynamic antibacterial effects. The synergy between BBR and SP enables continuous oxygen production that mitigates biofilm-associated hypoxia, a condition that enhances bacterial resistance. Additionally, the chlorophyll of SP can be activated by laser irradiation to produce ROS, which complement the bactericidal action by damaging bacterial membranes and DNA. In diabetic mouse models, wounds treated with the gel under laser irradiation showed significant reductions in bacterial colonies, improved tissue regeneration marked by angiogenesis, and reduced inflammatory cytokines.

PDT uses light-activated photosensitizers to generate ROS, effectively eradicating bacteria. Nanomaterials such as CDs, liposomes, and microorganism-derived platforms demonstrate exceptional potential in PDT, producing excessive ROS under NIR laser irradiation to damage bacteria. However, challenges remain in optimizing the photodynamic efficiency of nanomaterials under low-oxygen conditions, ensuring continuable ROS production, and balancing antibacterial activity with tissue biocompatibility. Developing platforms that overcome hypoxia, enhance biofilm penetration, and synergize PDT with other therapeutic modalities show great potential in enhancing the overall outcomes.

### 2.4 Designing nanomaterials for PTT

By converting light energy into localized heat, PTT can effectively disrupt bacterial cells, and stimulate tissue regeneration. Various nanomaterials, including PDA-based nanomaterials, CDs, melanin-based nanomaterials, Au NPs, CuO/CuS NPs, MoS_2_ nanosheets, metal-phenolic NPs, MXenes, and microbial-based nanomaterials have emerged as promising platforms for managing bacterial infections and promoting wound healing.

PDA is a synthetic melanin-like polymer known for its strong photothermal conversion efficiency, attributed to its extensive conjugated π-electron system. This unique structure enables PDA to absorb NIR light and convert it into heat, making it an ideal material for photothermal therapy applications. For example, to address the complexities of chronic wounds, a functionalized nanocomposite (mPDA@DFO@CP-SNO) was created by combining mesoporous PDA (mPDA) loaded with deferoxamine (DFO) and a chitosan-graft-third generation poly(amidoamine) polymer functionalized with S-nitrosothiol groups (CP-SNO) [96]. Upon NIR laser irradiation, the nanocomposite exhibited mild-temperature PTT (MPTT) and controlled release of nitric oxide (NO) and DFO, which synergistically eradicated bacteria and biofilms. Moreover, the nanocomposite also displayed anti-inflammatory and wound-healing properties, promoting wound regeneration by upregulating hypoxia-inducible factor (HIF)-1α and VEGF. In addition, PDA can be easily modified on various NPs to endow them with PTT ability. For example, a Cu-doped mesoporous silica coated with PDA and glycol chitosan (MCS@PDA@GCS) was developed for targeted “hot ion therapy” to treat infected wounds [97]. Copper ions can enhance the proliferation and angiogenic differentiation of human umbilical vein endothelial cells (HUVECs), thereby accelerating wound healing. Upon NIR light irradiation, the NPs generated heat and released copper ions, creating a “hot ions effect” that efficiently inhibited bacterial growth, including MRSA and *E. coli*. This effect not only directly killed bacteria but also stimulated macrophages to polarize into M1 phenotype, boosting the immune response against infections. Additionally, the mild photothermal effect and copper ion release promoted endothelial cell migration and tube formation, stimulating angiogenesis and facilitating wound healing.

**Fig. 6:**
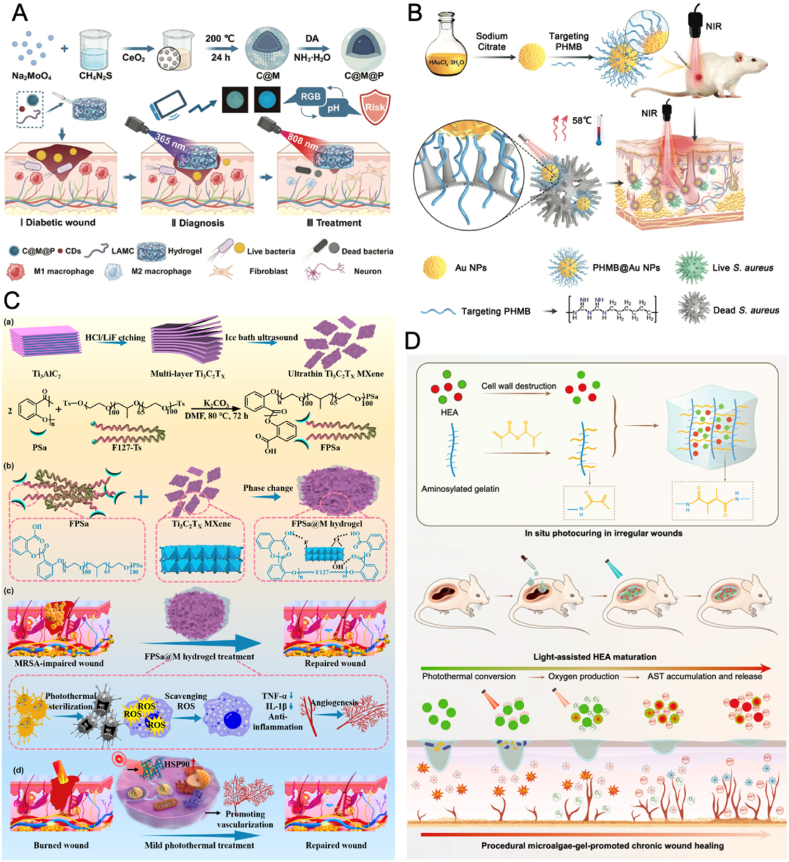
(A) Schematic illustration of the synthesis of LAMC/CD-C@M@P hydrogel and its diagnostic and therapeutic applications for infected-diabetic wound healing [99]. Copyright 2024, Wiley-VCH. (B) Schematic illustration of the fabrication of PHMB@AuNPs, and the in vivo antibacterial mechanism for promoting infected wound healing [37]. Copyright 2022, Wiley-VCH. (C) Schematic illustration of the fabrication of FPSa@M hydrogel for treating MRSA*-*infected burn wounds [117]. Copyright 2024, Elsevier. (D) Schematic illustration of the preparation of HEA@Gel and its mechanism in the management of wounds [120]. Copyright © 2024, Springer Nature.

In addition to PDT, CDs are also widely applied as photothermal agents for PTT [[85], [86], [87]]. To enhance the photothermal properties of CDs, Liu et al. doped iron into CDs to obtain ultrasmall Fe-CDs nanozymes with enhanced photothermal properties and photo-boosted enzyme-like activities for antibacterial therapy and wound repair [98]. The integration of iron significantly enhanced the generation of ROS and heat under NIR laser irradiation, effectively eliminating *S. aureus* and *E. coli*. In a mouse model with infected wounds further revealed that Fe-CDs promoted faster wound healing by stimulating neovascularization and reducing inflammation. The integration of NPs into hydrogels, nanofibers, or microneedles has shown great promise for advanced wound healing applications [[49], [50], [51], [52],^56^]. Wang et al. integrated pH-sensitive CDs and ceria oxide-molybdenum disulfide NPs with a polydopamine layer (C@M@P), into a lipoic acid-modified chitosan (LAMC) hydrogel (LAMC/CD-C@M@P) to monitor and promote diabetic wound healing (Fig. 6A) [99]. The CDs provided pH-sensitive fluorescence, enabling real-time monitoring of wound conditions via a smartphone, which facilitated early detection of bacterial infections by correlating pH variations (4–9) with the wound microenvironment. In addition, the C@M@P NPs in the hydrogel offered PTT antibacterial activity upon NIR light exposure, effectively killing bacteria such as *S. aureus* and *E. coli* by generating heat. Additionally, the hydrogel can mitigate oxidative stress, stimulate the polarization of macrophage towards the M2 phenotype, and enhance neural differentiation, thus fostering a conducive environment for healing.

Melanin is a natural pigment found in most living organisms, contributing to the coloration of skin, hair, eyes, and certain internal structures [100]. Its ability to absorb light and convert it into heat makes melanin a useful tool for selectively heating and killing bacterial cells [[101], [102], [103]]. For example, Qu et al. decorated natural cuttlefish ink nanoparticle (CINP) with zwitterionic polymer (ZP) as a dual-function agent (CINP@ZP) for photothermal and immunologic therapy against antibiotic-resistant bacteria [104]. CINP is a natural, biocompatible, and easily extractable resource, exhibiting innate photothermal properties derived from its rich melanin content and the CINP@ZP NPs efficiently eradicated MRSA and *E. coli* both in vitro and in vivo. Moreover, the CINP@ZP NPs were integrated into a thermosensitive Pluronic F127, which allowed for in situ gel formation (CINP@ZP-F127) and sustained release of NPs, showing enhanced antibacterial effects in mice with wound infections. Similarly, Luo et al. utilized melanin derived from black hair to synthesize self-adhesive microparticles (AMPs), which were embedded in a chitosan oligosaccharide-polyacrylic acid (CSO-PAA) hydrogel for wound healing [105]. The AMPs remained non-adhesive in dry conditions but became adhesive on wet surfaces through hydrogen bond-mediated adhesion, making them suitable for storage and on-demand applications. The melanin NPs within the AMPs scavenged ROS to mitigate oxidative stress and exhibited photothermal effects under NIR irradiation, accelerating tissue repair by promoting cellular metabolism and angiogenesis. Moreover, the AMPs potently inhibited the growth of *E.coil* growth, and in vivo testing confirmed the AMPs could adhere to wound sites and significantly expedite the healing process.

AuNPs have gained significant attention in antimicrobial applications due to their excellent biosafety, ease of functionalization, and outstanding photothermal efficiency [36,106]. A vehicle-free antimicrobial polymer hybrid AuNPs system (PHMB@AuNPs), which integrated the bactericidal and anti-biofilm functions of polyhexamethylene biguanide (PHMB) with the PTT capabilities of AuNPs, were developed for promoting wound healing in *S. aureus* infections (Fig. 6B) [37]. The synergistic effect of PHMB and AuNPs enhanced the photothermal bactericidal effect on *S. aureus*, inhibited biofilm formation, and rapidly cleared bacteria, promoting efficient wound healing in vivo. Moreover, PHMB@AuNPs promoted the transition of macrophage from the M1 phenotype to the M2 phenotype, facilitating inflammation resolution and tissue regeneration. In a mouse model with wound infections, PHMB@AuNPs rapidly eliminated bacteria in subcutaneous abscesses and infected wounds, accelerating the healing process with minimal toxicity to healthy tissues.

Cu-based NPs, such as CuO or CuS NPs, are well-known for their high photothermal conversion properties [39,107]. For example, Ye et al. developed CuO and AgO co-modified ZnO nanocomposites (CuO@AgO/ZnO NPs) for *S. aureus*-infected wound healing under NIR light [108]. CuO was selected for its PTT performance and ability to promote tissue regeneration, while AgO was incorporated due to its excellent antibacterial efficiency, which significantly enhanced the overall antimicrobial activity of ZnO NPs, especially when combined with NIR light, leading to high PTT stability and effectively eradicated *S. aureus* and *P. aeruginosa* in vitro.

The integration of metal ions with polyphenolic compounds has demonstrated significant potential in creating multifunctional nanomaterials for infected-wound healing applications [[109], [110], [111], [112], [113]]. For example, Qin et al. fabricated metal-phenolic nanozymes (TA-Fe/Cu nanocapsules) for addressing bacterial-infected diabetic wounds [114]. TA, a polyphenol with innate antibacterial properties, forms hydrogen bonds with bacterial peptidoglycan via its polyphenolic structure, enabling effective bacterial capture. Additionally, Cu^2^⁺ play crucial roles in promoting angiogenesis, thus supporting tissue regeneration during wound healing. Subsequently, the TA-Fe/Cu nanocapsules can not only generate heat upon exposure to NIR light to disrupt bacterial cell membranes, but also demonstrate ROS scavenging capabilities to reduce inflammation and fostering a more favorable healing environment. Both in vitro and in vivo studies demonstrated that the nanocapsules achieved up to 99 % bacterial eradication against MRSA and *E. coli*, while significantly improving wound closure in diabetic mice with infected wounds.

Two-dimensional (2D) nanomaterials, such as MXenes, molybdenum disulfide (MoS_2_) nanosheets and black phosphorus nanosheets (BPNs), have gained significant attention in antibacterial applications due to their unique physicochemical properties and multifunctional capabilities. Among them, MXenes is a class of 2D transition metal carbides, nitrides, or carbonitrides, possess unique features such as high electrical conductivity, strong NIR photothermal activity, hydrophilicity, and excellent biocompatibility [115,116]. Inspired by these advantages, Ma et al. incorporated Ti_3_C_2_T_X_ MXene with a poly(salicylic acid)-Pluronic F127-poly(salicylic acid) matrix to fabricate MXene-based hydrogel (FPSa@M) for treating wounds infected with MRSA and burn wounds (Fig. 6C) [117]. The FPSa@M hydrogel possessed exceptional photothermal conversion capabilities, effectively raising temperatures to kill *E. coli*, *S. aureus*, and MRSA. Additionally, the hydrogel could scavenge ROS, downregulate pro-inflammatory cytokines like TNF-α and IL-1β, and promote cellular activities essential for wound repair, such as endothelial cell migration and tubule formation. In MRSA-infected and burn wound mice models, when applied with NIR stimulation, the FPSa@M hydrogel not only eradicated bacterial infections but also enhanced tissue regeneration, resulting in faster wound healing, decreased inflammation, and increased formation of skin structures such as hair follicles and blood vessels. BPNs stand out for their strong photothermal effects, high biodegradability, and ability to release phosphate ions during degradation, promoting not only antibacterial action but also tissue regeneration and remineralization. For example, a BNP loaded hydrogel achieved up to 99 % bacterial eradication against *Streptococcus mutans* and *Streptococcus sanguinis* under NIR irradiation [118]. MoS_2_ NSs possess exceptional photothermal conversion efficiency, broad NIR absorption range, excellent stability under laser irradiation, and favorable biocompatibility, making them ideal candidates for PTT in antibacterial applications. A recent study by Qiu et al. immobilized pectinase (Pec) on MoS_2_ NSs through ε-poly-L-lysine (PLL) as an innovative nanoplatform (Pec@PLL-MoS_2_) for infected wound management [119]. Pec enabled programmed degradation of EPS, dismantling the biofilm matrix that protects bacteria. Under 808 nm laser irradiation, MoS_2_ generates localized heat, enhancing the enzymatic activity of Pec and improving biofilm penetration and antibacterial efficacy. Subsequently, Pec@PLL-MoS_2_ eradicated up to 89.9 % of *E. coli* and 81.1 % of *S. aureus* biofilms within 5 min, reducing bacterial survival rates to as low as 0.1 %. In vivo experiments in a biofilm-infected wounds mouse model further confirmed its ability to completely eradicate biofilm infections, reduce inflammation, and accelerate tissue regeneration.

*Haematococcus* (HEA) cells exhibit strong photothermal effects under high light intensity, while show oxygen producing ability under low light intensity. By virtue of this characteristic, Kang et al. incorporated live HEA cells into GelMA (gelatin methacryloyl) to obtain a programmed microalgae-gel (HEA@Gel) for chronic diabetic wound healing (Fig. 6D) [120]. Under high light intensity, the green HEA cells (GHEA) exhibit strong photothermal effects that eradicate bacteria by increasing the wound temperature, facilitating surface disinfection, which was effective in significantly reducing bacterial presence in wounds on diabetic mice. By modulating the light intensity to a lower level, GHEA shifts its function to oxygen production through photosynthesis, which enhanced cellular processes such as fibroblast proliferation and angiogenesis, promoting tissue regeneration. Over time or under continuous light exposure, the GHEA cells accumulate astaxanthin (AST), transitioning into red HEA cells (RHEA). AST, known for its potent antioxidant properties, reduced oxidative stress by scavenging excess ROS, and supported macrophage polarization to the M2 phenotype. Subsequently, the HEA@Gel demonstrated superior efficacy in enhancing wound healing, reducing bacterial infection, and promoting vascularization in vivo.

PTT leverages light-activated nanomaterials to generate localized heat, effectively disrupting bacterial membranes. Nanomaterials such as PDA offer strong photothermal conversion with additional functionalities like ROS scavenging and immune modulation. Continued research into PTT applications may emphasize optimizing thermal control and balancing antibacterial efficacy with biocompatibility to avoid damage to healthy tissues. And combining PTT with oxidative stress modulation or immune-regulating strategies could further its impact in wound healing.

### 2.5 Designing nanomaterials for SDT

**Fig. 7:**
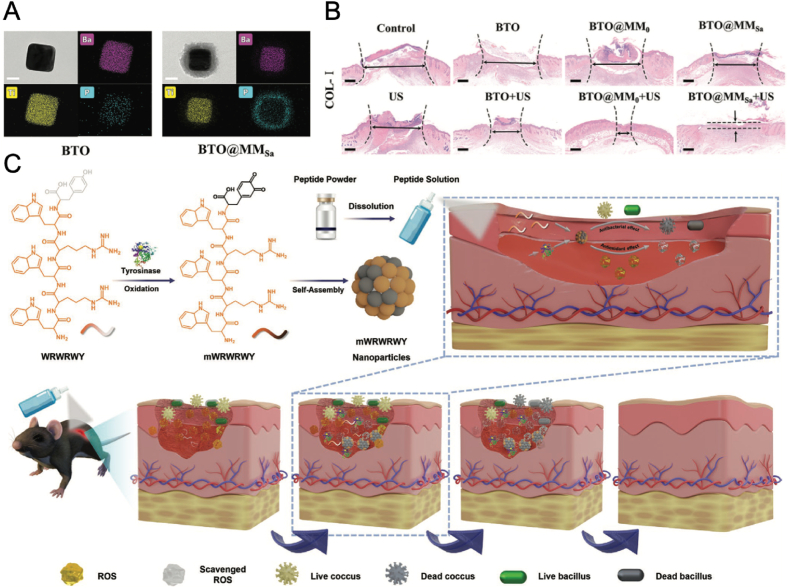
(A) TEM images and elemental mappings of Ba, Ti, and P of BTO and BTO@MM_Sa_ (Scale bars = 50 nm). (B) Hematoxylin and eosin (H&E) staining of the skin tissues after various treatments on day 12 indicated the BTO@MM_Sa_ + US treatment can significantly decrease the granulation tissue width (Scale bars = 200 μm) [123]. Copyright 2024, Wiley-VCH. (C) Schematic illustration of the in situ synthesis of mWRWRWY NPs and their applications in promoting infected-wound healing [46]. Copyright 2023, Wiley-VCH.

SDT is an emerging antibacterial strategy that utilizes ultrasound to activate sonosensitizers, resulting in the production of ROS to kill bacteria. Unlike traditional antibacterial methods, SDT offers the advantages of deep tissue penetration, spatial selectivity, and minimal invasiveness, making it particularly suitable for treating infected wounds. Various nanomaterial-based sonosensitizers, such as inorganic nanoparticles (e.g., TiO_2_, ZnO, barium titanate (BaTiO_3_, BTO)) and nanocarriers encapsulated with small molecule sonosensitizers have attracted significant attention in SDT for antibacterial applications due to their stability, efficient ROS generation, and unique physical properties [121]. Among these sonosensitizers, BTO is a piezoelectric material with a non-centrosymmetric crystal structure. Upon exposure to ultrasound waves, BTO generates a localized electric field and surface potential, which in turn prompts the generation of ROS [122]. By virtue of the efficient SDT effect of BTO, Qian et al. developed a bioinspired sonodynamic nano spray system for targeted treatment and accelerated wound healing [123]. The system utilizes macrophage membranes pre-activated by *S. aureus*, which are then coated onto ultrasound-triggered piezocatalytic BTO NPs, forming a novel targeted delivery nanosystem called BTO@MM_Sa_ (Fig. 7A). The BTO@MM_Sa_ nanosystem efficiently targets infected areas, generates ROS under ultrasound irradiation to kill bacteria, and promotes wound healing. Prokaryotic RNA-seq transcriptomics revealed that the antibacterial ability of BTO@MM_Sa_ with ultrasound is mainly due to the disruption of bacterial membrane function and suppression of substance metabolism, biosynthetic processes, and energy metabolism. The wound-healing properties of the nano spray were further validated by its excellent biocompatibility and its ability to enhance fibroblast migration and promote collagen production (Fig. 7B).

Enhancing the ROS generation through heterojunction engineering is promising to enhance the SDT effect of sonosensitizers. Qian et al. developed a novel sonodynamic-nanocatalytic platform using graphene quantum dots (GQDs) loaded on TiO_2−x_ microspheres (GQDs/TiO_2−x_) [124]. The presence of oxygen vacancies in TiO_2−x_ increases Ti^3+^ sites, which not only enhance ROS generation through Ti^3+^/Ti^4+^-mediated enzyme-like activities but also improve electron-hole pair separation. The incorporation of GQDs further amplifies SDT activity by promoting carrier transfer, while GQDs themselves act as nanoantibiotics by inhibiting bacterial topoisomerase I. This synergistic system exhibited superior antibacterial activity against multidrug-resistant pathogens, significantly reducing biofilm formation and promoting wound healing in animal models. Another innovative approach involves Li-doped ZnO nanorods incorporated into poly(L-lactic acid) (PLLA) microfibers (ZnLiPOI) [125]. Li doping enhanced the piezoelectric and SDT properties of ZnO by increasing the bandgap and generating a robust electric field under ultrasound stimulation. These microfibers exhibited spatiotemporal therapeutic effects, effectively eliminating over 94 % of *S. aureus* within 15 min of ultrasound exposure. Beyond the antibacterial phase, the microfibers mitigate oxidative stress by capturing ROS, facilitating macrophage polarization, and restoring mitochondrial function. The release of bioactive Zn^2+^ and Li ^+^ ions during later healing stages promotes cell recruitment and neuro-vascularization, demonstrating a comprehensive strategy for infected wound management.

SDT employs ultrasound to activate sonosensitizers, generating ROS for bacterial eradication. With advantages like deep tissue penetration and spatial precision, SDT is particularly promising for infected wound management. Advancing SDT technologies might involve refining sonosensitizer design to improve biocompatibility and ROS generation efficiency and combining SDT with multifunctional therapeutic platforms could offer more targeted and effective solutions for infected wounds.

### 2.6 Designing nanomaterials with positive surface charge

Nanomaterials with positive surface charge can electrostatically bind to negatively charged bacterial membranes, disrupting the membrane and causing bacterial death [23,126]. Chitosan (CS) is a natural polysaccharide with positive charge derived from chitin through a deacetylation process, exhibiting excellent compatibility, a natural propensity to combat microbial growth [[127], [128], [129]]. Inspired by the intrinsic antibacterial properties and high biocompatibility of CS, Zhan et al. designed a multifunctional nanofiber dressing, CS@PLCL/DWJM@Cu, with a core-shell structure incorporating CS, Cu ions, and decellularized Wharton's jelly matrix (DWJM) [43]. In the initial phase, the CS shell releases its content rapidly, exerting strong antibacterial effects, which are vital for the early stages of wound healing. Then, Cu ions from the core are gradually released, encouraging the production of growth factors such as VEGF and PDGF, which stimulate endothelial cell migration and angiogenesis. Meanwhile, DWJM, which is rich in extracellular matrix proteins and growth factors, supports cellular adhesion and proliferation, enhances collagen deposition, and provides a scaffold that replicates the natural wound healing environment, facilitating the transition from repair to regeneration.

In addition to perform as PTT and/or PDT agents, CDs designed with positively charge can also be directly applied as antibacterial agents without external stimulations. For example, Qu et al. fabricated a positively charged CDs synthesized from p-phenylenediamine and polyethyleneimine, exhibiting strong antibacterial and antioxidant properties while maintaining excellent biocompatibility [44]. The CDs effectively killed *S. aureus*, scavenged excess free radicals, reduced oxidative stress, and facilitated the transition from inflammation to the proliferation phase during wound healing. In mice with skin infection, applying CDs via simple drop or spray onto infected skin wounds significantly promoted the healing process without notable side effects.

Antimicrobial peptides (AMPs), key components of the innate immune system found in microorganisms, plants, and animals, are emerging as promising alternatives to conventional antibiotics [130]. Particularly, cationic AMPs represent a group of positively charged peptides known for their wide-ranging antibacterial properties [131]. Xing et al. designed a natural AMPs-containing chiral gel dressing (HA-LM2R-MR), which includes a cationic hexapeptide RWRWRW derivative, for treating diabetic wounds complicated by multidrug-resistant bacterial infections and advanced glycation end products (AGEs) [45]. The chiral nature of the dressing allowed for stereoselective interactions with AGEs, facilitating their removal from the wound site, while the cationic hexapeptide selectively disrupted the negatively charged biofilms and membranes of bacteria, demonstrating a potent antibacterial effect without harming host cells. The dressing also promoted angiogenesis by upregulating VEGF and OPA1 expression in human umbilical vein endothelial cells. In a mouse model with MRSA infected diabetic wounds, the HA-LM2R-MR dressing achieved superior healing outcomes, reducing the recovery period from 21 days to 14 days compared to standard clinical treatments.

Similarly, Teng et al. created a novel AMP, Trp-Arg-Trp-Arg-Trp-Tyr (WRWRWY), which self-assembles into NPs (mWRWRWY) at the wound site, offering an innovative approach to infected wound healing (Fig. 7C) [46]. In WRWRWY, tryptophan (W) provided lipophilicity for membrane interaction, while arginine (R) offered a high pKa value for stable ionic interactions, which together enhanced the antimicrobial activity of the peptide. Moreover, the tyrosine (Y) introduced in this peptide can be oxidized by tyrosinase, an enzyme naturally present in human skin, thus triggering the formation of NPs with increased positive charge density. The high local concentrations of AMPs at the wound site enhanced the peptides' stability and antibacterial efficiency against both *E. coli* and *S. aureus*. In addition, the resulting NPs exhibited a melanin-like structure, providing efficient antioxidant properties by scavenging ROS. In mouse model with *S. aureus* Infected wound, the in situ formed mWRWRWY NPs can efficiently heal infected wounds and significantly reduce ROS levels.

Positively charged nanomaterials, such as chitosan-based particles and AMPs, leverage electrostatic interactions to disrupt bacterial membranes. Innovations like AMP self-assembling nanoparticles combine strong antibacterial effects with wound healing functionalities, such as angiogenesis. Key challenges include ensuring stability in complex environments and minimizing host cell damage. Scaling up production and ensuring the versatility of these platforms in diverse wound environments will be essential for clinical application.

### 2.7 Designing nanomaterials with novel antimicrobial mechanism

**Fig. 8:**
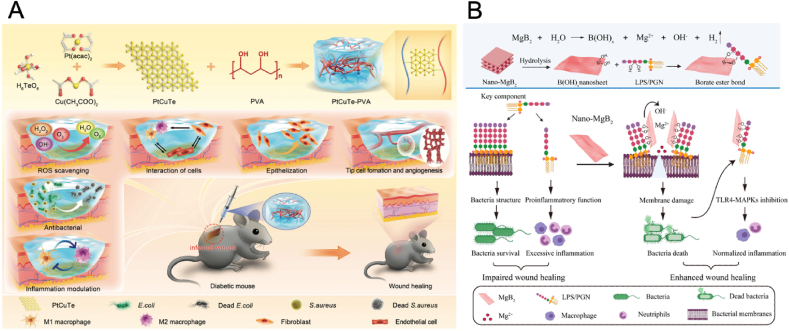
(A) Schematic illustration of the synthesis of PtCuTe nanosheets with robust ROS scavenging activities and ROS-independent antibacterial properties, thus promoting the infected diabetic wounds healing [132]. Copyright 2024, Wiley-VCH. (B) Schematic illustration of the synthesis of Nano-MgB_2_ and the boron-trapping strategy for bacteria-infected wound healing [133]. Copyright 2022, Springer Nature.

With the development of nanotechnology, various new nanomaterials have been constructed and new anti-bacteria mechanisms have been revealed. For example, Guo et al. designed novel PtCuTe nanosheets with robust ROS scavenging properties and ROS-independent antibacterial properties for treating infected diabetic wounds (Fig. 8A) [132]. The nanosheets significantly reduced bacterial loads in a concentration-dependent manner, demonstrating strong antibacterial effects against *S. aureus* and *E. coli*. This antimicrobial action was found to be primarily driven by bacterial membrane damage and inhibition of flagellar motion caused by Te, rather than through ROS production or DNA damage. Besides, the nanosheets exhibited enhanced ROS-scavenging capabilities compared to conventional PtCu materials due to the robust electronic interactions between Te and other constituent elements. Furthermore, PtCuTe nanosheets supported wound healing by stimulating vascular tube formation, promoting macrophage polarization to the M2 phenotype, and improving fibroblast migration.

The two major challenges in treating non-healing wounds are bacterial infections and the resulting inflammation caused by the release of lipopolysaccharide (LPS) and peptidoglycan (PGN) from dead bacteria. Traditional methods, which primarily focus on eradicating bacteria, often fail to manage the inflammation triggered by these bacterial components. Meng et al. developed an innovative reactive metal boride NPs (MB NPs), with diverse and complex bonding characteristics, especially the relatively weak B–M (boron-metal) ion bonds, for targeting wound infections and inflammation [133]. The MB NPs, such as Nano-MgB_2_, can hydrolyze to produce boron dihydroxyl groups and metal cations, creating an alkaline microenvironment that facilitates esterification reactions between boron dihydroxyl groups and LPS or PGN. This reaction forms stable borate ester bonds, effectively disrupting bacterial membranes and neutralizing inflammation caused by bacterial components (Fig. 8B). At a low concentration (12.5 μg/mL), Nano-MgB_2_ outperformed conventional MgB_2_ powders and exhibited antibacterial efficacy against *P. aeruginosa* comparable to that of several antibiotics, including gentamicin, amikacin, and ciprofloxacin. In a mouse model with *P. aeruginosa*-infected wounds, Nano-MgB_2_ accelerated wound healing by reducing bacterial growth and inflammation.

Metal-phenolic NPs combines the advantages of metal ions and polyphenols, which synergistically enhance the antibacterial effect [[109], [110], [111], [112], [113]]. Yu et al. developed Cu^2+^-based metal-phenolic network NPs (am-MPN NPs) using (−)-epigallocatechin gallate (EGCG, a representative tea polyphenol) for antibacterial therapy and wound healing [134]. The NPs were synthesized using a one-step assembly process that involved diethyldithiocarbamate (DEDCT, an FDA-approved antidote for heavy metal poisoning), EGCG, and Cu^2^⁺. They demonstrated that the Cu^2+^-based am-MPN NPs effectively disrupted bacterial cell walls and promoted ROS production. Moreover, the phenolic groups on EGCG are prone to be oxidized by Cu^2+^ to form electron-deficient semiquinones, which can conjugate with the sulfhydryl residues on proteins to form quinoproteins, leading to bacterial death. Subsequently, Cu^2+^-based am-MPN NPs were effective against multidrug-resistant bacteria, such as MRSA, successfully inhibiting bacterial biofilms and promoting the healing of MRSA-infected skin wounds.

Emerging nanomaterials, such as PtCuTe nanosheets and MB NPs, demonstrate novel antimicrobial mechanisms, including ROS-independent bacterial membrane disruption and inflammation modulation. These innovations address limitations of traditional methods and enable simultaneous infection control and tissue repair. Building on these innovations, future work could explore integrating such novel mechanisms into multifunctional platforms. Addressing biocompatibility and scalability challenges will be key to transitioning these technologies into practical applications.

### 2.8 Designing nanomaterials with multi-antibacterial mechanisms

The integration of multiple antibacterial mechanisms into single nanoplatform offers significant advantages for treating wound infections [135,136]. By leveraging the synergistic effects of multiple mechanisms, these multifunctional nanomaterials can be tailored to respond to specific wound microenvironments, enabling precise and targeted antibacterial action. Moreover, these nanomaterials can also be designed for promoting tissue regeneration through the controlled release of bioactive ions or by modulating inflammation, making them a powerful and versatile platform for managing complex wound infections.

#### 2.8.1 Designing nanomaterials with PTT/PDT and ion release abilities

PTT combined with ion release represents an innovative dual-functional strategy for wound management. AgNPs, apart from releasing Ag^+^ ions with potent antibacterial effects, also demonstrate remarkable photothermal conversion efficiency, which makes them particularly suitable for addressing bacterial infections. For example, Liu et al. developed gallic acid functional AgNPs (GA-Ag NPs) embedded carrageenan hydrogel to serve as a photothermal antibacterial platform [137]. The GA-Ag NPs can not only release Ag^+^, but also exhibit good photothermal conversion efficiency (48.7 %), generating heat under 808 nm NIR light exposure and synergistically inducing bacterial death. Consequently, the hydrogel exhibits broad-spectrum sterilization properties against both *E. coli* and *S. aureus*, with in vivo tests confirming its antibacterial effect and wound healing properties. Similarly, Cao et al. decorated Ag-bismuth NPs on silica (Ag-Bi@SiO_2_ NPs), which exhibited enhanced Ag^+^ ions release under PTT, showing synergistic therapy against skin infections, particularly MRSA [138]. These nanoparticles exploited the photothermal activity of bismuth to boost Ag^+^ ion release, significantly improving their antibacterial action. When irradiated with NIR laser, the NPs substantially reduced MRSA cell populations and biofilm biomass. Mouse studies confirmed the NPs’ therapeutic potential, with up to 95.4 % of bacteria elimination and rapid abscess resolution.

#### 2.8.2 Designing nanomaterials with PTT and CDT abilities

**Fig. 9:**
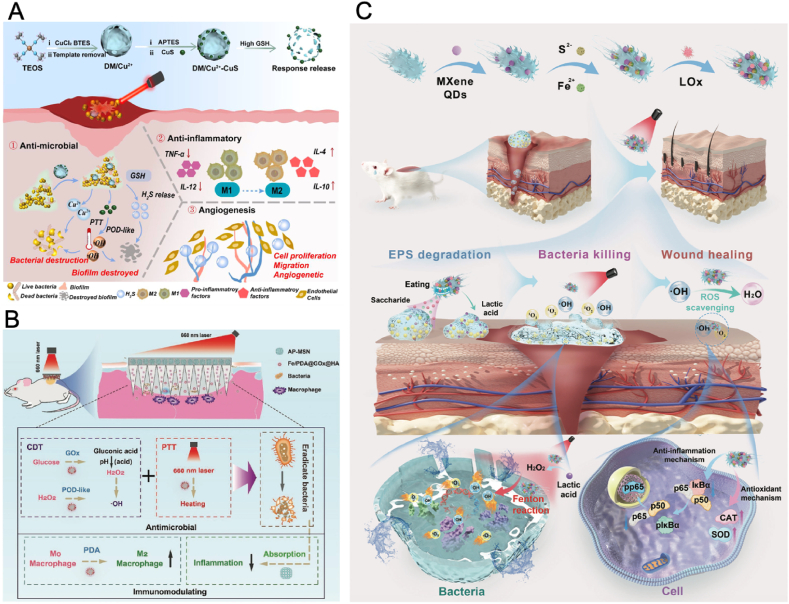
(A) Schematic illustration of the preparation of DM/Cu^2+^-CuS and its antibacterial mechanism [139]. Copyright 2024, Elsevier. (B) Schematic illustration of the therapeutic mechanism of PFG/M MN for infected-wound healing [141]. Copyright 2023, Wiley-VCH. (C) Schematic illustration of the fabrication of P-bioHJ and its mechanisms, including antibiofilm, antioxidant, and anti-inflammatory actions, for treating biofilm-infected wounds [143]. Copyright 2024, Wiley-VCH.

Combining PTT with CDT within a single nanoplatform offers a powerful dual-functional approach for tackling bacterial infections, especially in biofilm-laden or multidrug-resistant environments. Cu is vital for redox reactions in the human body, and Cu-based NPs have diverse biological activities, including multiple enzyme-like activities, and photothermal property [39]. Zeng et al. synthesized Cu-containing NPs-loaded silicene nanosheets (Cu_2.8_O@silicene-BSA) that exhibited triple enzyme-mimic activities and photothermal effects for healing drug-resistant bacteria-infected wounds [40]. The nanosheets mimic the activities of POD, OXD, and CAT, enabling the production of ROS and oxygen for CDT. Additionally, the nanosheets displayed strong NIR absorption and a photothermal conversion efficiency of 23.8 %, allowing it to function as a photothermal agent for PTT while simultaneously boosting their enzyme-mimic functions for enhanced CDT. Consequently, the nanosheets eliminated MRSA with over 99 % antibacterial efficiency in vitro and 97 % in vivo. Likewise, Li et al. loaded Cu^2+^ and CuS into dendritic mesoporous organosilica NPs (DM/Cu^2+^-CuS), which could release Cu^2+^ ions, CuS, and hydrogen sulfide (H_2_S) gas in response to the high GSH concentrations in biofilm environments (Fig. 9A) [139]. Under 808 nm laser irradiation, CuS exhibited PTT and CDT properties, generating ROS and H_2_S gas. H_2_S enhanced macrophage polarization to the M2 phenotype, disrupted extracellular DNA in biofilms, and amplified the effects of PTT and CDT. Additionally, the GSH-depleting action of the DM particles improved ROS efficiency and strengthened antibiofilm effects. The integration of PTT, CDT, and gas therapy achieved high antibacterial efficiency against *S. aureus* and *E. coli* in vitro and demonstrated significant safety and effectiveness in vivo.

The CDT efficiency may be hindered due to the hypoxia and limited H_2_O_2_ availability in the wound microenvironment. To overcome this challenge, a pH-switchable nanozyme platform (FeCP/ICG@CaO_2_) was created by integrating a ferrocenyl coordination polymer (FeCP) nanozyme with indocyanine green (ICG) and calcium peroxide (CaO_2_) for treating skin tumor wounds infected with drug-resistant bacteria [140]. In this system, CaO_2_ generated both H_2_O_2_ and O_2_ upon contact with water, facilitating ROS production for bacterial killing and alleviating hypoxia in wound tissues to promote healing. FeCP is a ferrocenyl coordination polymer, exhibiting POD-like activity in acidic conditions to enhance ROS production, and switched to CAT-like activity at neutral pH to decompose H_2_O_2_, preventing oxidative damage and promoting wound healing. Meanwhile, upon NIR laser irradiation, ICG generated heat, further amplifying the enzymatic activity of FeCP. The FeCP/ICG@CaO_2_ achieved 99.8 % antibacterial efficacy against MRSA and kanamycin-resistant *E. coli* (KREC) in vitro. In mice with MRSA-infected skin tumor wounds, FeCP/ICG@CaO_2_ not only significantly accelerated wound closure but also promoted tissue regeneration, reduced inflammation, and enhanced angiogenesis in both normal and MRSA-infected skin tumor wounds.

To achieve sufficient drug delivery into deep dermis for wound healing, microneedle (MN) systems integrated with NPs have emerged as an effective strategy. Li et al. decorated iron oxide NPs with photothermal agent PDA, which were then modified with glucose oxidase (GOx) and hyaluronic acid (HA), and loaded into MN tips, with amine-modified mesoporous silica NPs (AP-MSNs) in the bases [141]. Under the low pH and high GSH conditions of infected wounds, the MNs decomposed to release GOx and iron ions, facilitating CDT by producing ROS to kill bacteria. Additionally, the photothermal properties of PDA converted light energy into heat, creating a synergistic effect with CDT to eradicate bacteria without inducing drug resistance. In addition, the AP-MSNs in the MN bases further modulated the immune response by adsorbing pro-inflammatory factors such as free nucleic acids, promoting a microenvironment conducive to wound healing (Fig. 9B).

#### 2.8.3 Designing nanomaterials with CDT, PTT, and PDT abilities

The integration of CDT, PTT, and PDT within a single nanoplatform enables a tailored approach to tackling drug-resistant bacterial infections. By leveraging inherent catalytic or structural properties of nanomaterials like palladium NPs (Pd NPs) [142], MXene [143], or Cu-based NP [144], various innovative designs have been developed to enhance antibacterial and regenerative effects.

Pd NPs exhibit multi-enzyme-like activities and photothermal efficiency, making them valuable in antibacterial and antitumor therapies, drug delivery, and imaging applications [145]. Li et al. developed palladium nanosheets (PdNS) with intrinsic bactericidal properties and external light-triggered antibacterial effects for tackling wound infected with multidrug-resistant (MDR) bacteria [142]. On the one hand, PdNS possessed basal bactericidal activity via a “nanoknife” effect, mechanically disrupting bacterial cell membranes, and POD-like catalytic activity, without light exposure. When exposed to NIR light, PdNS showed enhanced antibacterial effects via photothermal and photodynamic mechanisms, generating heat and ROS to further damage bacterial membranes and disrupt metabolism. The addition of H_2_O_2_ activated the CAT-like properties of PdNS, further boosting ROS production and enhancing the photodynamic effect. In mouse models with *S. aureus* or *P. aeruginosa* infected wounds, the combination of PdNS, H_2_O_2_, and NIR light significantly reduced bacterial load at the wound site, achieving bactericidal rates greater than 92.7 % and nearly complete wound recovery within eight days post-treatment.

Degrading the EPS is vital for combating bacyeria, Qin et al. developed an engineered probiotic bio-heterojunction (P-bioHJ) by combining *Lactobacillus rhamnosus* (LG) with MXene (Ti_3_C_2_) quantum dots (MQDs)/FeS and Felactate oxidase (LOx), which can degrade the EPS and leverage PTT to eliminate bacterial cells (Fig. 9C) [143]. LG metabolized saccharides to lactic acid in the EPS of biofilms, breaking down the structural matrix that protects bacteria from external agents, which allowed the subsequent penetration of antimicrobial agents. Furthermore, the LOx catalyzed lactic acid to generate H_2_O_2_ and Fe^2+^ converted H_2_O_2_ into highly toxic ·OH, and the PTT/PDT effect of MQDs/FeS were activated by NIR laser irradiation, inducing intensive oxidative stress to combat bacteria. Additionally, during wound healing, the P-bioHJ also reduced ROS levels and suppressed the NF-κB inflammatory pathway, accelerating wound healing.

Developing platforms to not only eradicate bacteria but also actively support tissue regeneration is promising to bridge the gap between antibacterial efficacy and wound healing in complex infected environments. Zhou et al. integrated tannic acid (TA)-Fe complex with CuS NPs (CuS@TA-Fe NPs), and embedded them in hydrogel composed by gelatin quaternary ammonium salt and dialdehyde alginate, which retained the CDT, PTT, and PDT properties of CuS@TA-Fe NPs and endowed them with anti-inflammatory and antioxidant functions [144]. The NPs exhibited POD-like activity to produce ROS for CDT, and their superior photothermal conversion efficiency and ROS generation under NIR light further enhanced the therapeutic efficiency, achieving bacterial killing rates of nearly 99 % against *E. coli* and *S. aureus* after 5 min of NIR irradiation. Additionally, the incorporation of TA endows the system with anti-inflammatory and antioxidant properties, creating a conducive environment for wound healing. In a rat model of *S. aureus*-infected wounds, the CuS@TA-Fe hydrogel significantly accelerated wound closure, reduced inflammation, and promoted collagen deposition and re-epithelialization.

#### 2.8.4 Designing nanomaterials with PTT and gas release abilities

Gas therapy has emerged as a powerful strategy in antibacterial treatment and wound healing, utilizing physiologically active gases such as NO and carbon monoxide (CO) [146]. These therapeutic gases effectively penetrate bacterial biofilms, disrupt metabolic processes, and modulate the wound microenvironment by reducing inflammation and promoting tissue regeneration. Unlike traditional antibiotics, gas therapy addresses biofilm-associated infections and enhances bacterial susceptibility to other treatments. When combined with PTT, gas therapy benefits from PTT-induced hyperthermia, which enhances gas release and amplifies antibacterial efficacy. For instance, Ma et al. developed a surface charge-adaptive NO nanogenerator (PDG@Au–NO/PBAM) that combines photothermal properties with NO generation [147]. This nanogenerator featured a core-shell structure, where AuNPs conjugated on cationic poly(dopamine-co-glucosamine) (PDG) NPs and the NO donor-conjugated AuNPs generate hyperthermia under NIR irradiation while releasing NO in a controlled manner, disrupting EPS matrix and eradicating drug-resistant bacteria. The adaptive surface charge mechanism enhances biofilm penetration, while the burst NO release under NIR irradiation amplifies the antibacterial effect, which not only kills bacteria effectively but also mitigates damage to surrounding tissues. In another example, Xu et al. reported a programmed gas release NPs (PB-SNP@SiO_2_, PSS NPs) for diabetic infected wound treatment [148]. This nanoplatform utilized Prussian blue (PB) NPs as the photothermal core, combined with a NO donor (sodium nitroprusside, SNP) for antibacterial therapy and a H_2_S-releasing silica shell for anti-inflammatory and pro-angiogenic effects. Upon NIR irradiation, the PB core generates hyperthermia and releases NO, eliminating bacterial biofilms and pathogens. Simultaneously, the H_2_S release triggered by the GSH-rich microenvironment alleviates inflammation, promotes macrophage polarization, and facilitates revascularization, significantly accelerating wound healing. Similarly, Mu et al. employed the MoS_2_ NSs to load NO donor (S-nitrosothiol-modified chitosan, SNO-CS) as a novel multimodal antibacterial nanoplatform (SNO-CS@MoS_2_) [149]. Upon NIR light irradiation, the MoS_2_ NSs generate localized heat, triggering the release of NO, which synergistically disrupts bacterial membranes, induces protein leakage, and impairs ATP synthesis, leading to efficient bacterial eradication. Residual SNO-CS@MoS_2_ continues to release trace amounts of NO, promoting fibroblast migration, proliferation, and angiogenesis, which accelerates tissue regeneration.

Beyond PTT, the combination of gas therapy with PDT also shows promise. For instance, a multifunctional nanoplatform (CGP) composed of guanidino-functionalized polymers and the photosensitizer chlorin e6 (Ce6) was developed to integrate antimicrobial PDT (aPDT) with NO gas therapy [150]. Upon laser irradiation, the nanoplatform generated ROS via Ce6 while simultaneously releasing NO through guanidino group oxidation, disrupting bacterial membranes, which was highly effective in eradicating *E. faecalis* biofilms and bacterial cells. In addition, Cai et al. further combined PTT, PDT, and CO gas therapy to address biofilm-associated bacterial infections [151]. They encapsulated the photosensitizer ICG and a CO-releasing molecule (MnBr(CO)_5_) within a peptide dendrimer-based nanogel to create ICG&CO@G3KBPY. Under laser irradiation, this nanoplatform synergistically generated heat, ROS, and CO, effectively disrupting biofilms, killing bacteria, and mitigating the inflammatory response caused by treatment. The release of CO enhanced biofilm penetration and further amplified antibacterial efficacy. In mouse models of catheter-related biofilm infections and subcutaneous abscesses, ICG&CO@G3KBPY successfully eradicated biofilms, reduced bacterial load, and promoted healing with minimal side effects.

#### 2.8.5 Designing nanomaterials with positive charge and PTT ability

Modulating the surface charge of nanomaterials for targeting and capturing negatively charged bacteria, while maintaining the PTT ability is a powerful dual mechanism for combating infections. Yu and colleagues designed self-assembled photothermal nanoparticles (MCC/CS NPs) composed of mono-carboxyl corrole (MCC) and CS to treat diabetic wounds infected with bacteria [47]. The MCC/CS NPs, with a core-shell structure, were formed via hydrogen bonding and π-π stacking interactions between MCC and CS. The positively charged chitosan enabled the NPs to capture negatively charged bacteria through strong electrostatic interactions, amplifying the antibacterial effects of CS. The MCC/CS NPs showed an impressive photothermal conversion efficiency of 66.4 % and exhibited strong antibacterial activity against *E. coli* and MRSA when exposed to NIR laser irradiation. In diabetic wound mice models, the MCC/CS NPs effectively killed MRSA, accelerated wound healing, and promoted angiogenesis, demonstrating excellent biocompatibility.

#### 2.8.6 Designing nanomaterials with PTD, PTT, and SDT abilities

Furthermore, integrating multiple external stimulations, such as light and ultrasound, into single platform is particularly effective for enhancing antibacterial efficiency. TiO_2_ NPs are highly promising antibacterial agents due to their unique photocatalytic properties [41]. When exposed to ultraviolet (UV) light, electrons in the valence band of TiO_2_ are excited to the conduction band, forming electron-hole pairs that interact with water and oxygen to produce abundant ROS. However, the excitation of TiO_2_ is restricted to UV radiation due to its broad bandgap, which ranges from 3.0 to 3.2 eV [152]. This limitation results in shallow tissue penetration and poses significant health risks, including cellular DNA damage and the potential for skin malignancies. To address these limitations, Cheng et al. synthesized mesoporous TiO_2_@polydopamine (mTiO_2_@PDA) NPs that integrate the benefits of PDT, PTT, and sonodynamic therapy (SDT) [42]. The mTiO_2_@PDA system leverages a ligand-to-metal charge transfer (LMCT) mechanism, enabling TiO_2_ to be activated by NIR light, which overcomes the limitations of TiO_2_ that traditionally requires harmful UV light for activation. Under NIR and ultrasound irradiation, mTiO_2_@PDA generates ROS and localized heat, which effectively disrupts bacterial membranes, inhibits biofilm formation, and induces cell death. In mouse model suffer *E. coli*-infected wound, mTiO_2_@PDA showed enhanced wound healing and reduced local ROS levels, indicating the therapeutic efficacy and biosafety of mTiO_2_@PDA NPs.

Integrating multiple antibacterial mechanisms, such as ion release, PTT, CDT, and gas therapy, into single platforms enables precise and synergistic effects. Multifunctional designs, such as CDT and PTT combinations, enhance ROS production and combat multidrug-resistant bacteria. Efforts to tailor multifunctional platforms for specific microenvironmental conditions could significantly enhance their adaptability and demonstrating their performance in clinically relevant settings will further support their transition into therapeutic applications.

## 3 Antibacterial drug delivery systems: Nanocarriers and beyond

The discovery of new antibiotics has significantly slowed, while the prevalence of resistant pathogens continues to rise at an alarming rate. Nanocarriers offer an innovative solution to this growing problem, enabling the re-engineering of traditional antibiotics with enhanced properties such as targeted delivery, multifunctionality, and controlled release mechanisms. Controlled-release nanocarriers ensure sustained antibacterial effects by maintaining effective drug concentrations at the infection site while minimizing systemic toxicity, extending therapeutic window and reducing the frequency of administration. Additionally, stimuli-responsive systems, triggered by environmental cues such as pH and ROS, allow for localized and on-demand release, further enhancing therapeutic efficacy and reducing off-target effects. These advancements not only address the limitations of conventional treatments but also hold significant potential in overcoming biofilm-related challenges and mitigating the emergence of antibiotic resistance (Table 2).

**Table 2:** Summary of antibacterial drugs loaded on nanocarriers for infected wound healings.

Nanosystems	Antibacterial drugs	Nanocarriers	Antibacterial spectrum	In vivo applications	Ref
GaMOF NPs	Gentamicin (Gen)	GaMOF NPs	*E. coli,* and *S. aureus*	*S. aureus*-infected wounds	[153]
PTM composites	Benzalkonium chloride (BC)	PCN-224	*E. coli*, *S. aureus*, and MRSA	MRSA-infected wounds	[154]
MPH NPs	Polymyxin B (PMB)	MIL-100	*S. aureus*, *E. coli*, and MRSA	*S. aureus*-infected wounds	[155]
Ag_2_S@ZIF-Van	Vancomycin (Van)	ZIF-8	*S. aureus*	*S. aureus*-infected wounds	[156]
GOx@Fe_x_S_y_/AZM	Azithromycin (AZM)	GOx@Fe_x_S_y_	*S. aureus*	*S. aureus*-infected wounds	[157]
AB@LRM	Amikacin (AM)	Black phosphorus quantum dots (BPQDs)	*P. aeruginosa*	*P. aeruginosa*-infected wounds, and bacterial pneumonia	[158]
CIP-Ceria-PVs	Ciprofloxacin (CIP)	PCL-b-PGA	*E. coli,* and *S. aureus*	*S. aureus*-infected wounds	[159]
DNGs-CIP	CIP	Dendritic nanogels (DNGs)	*E. coli,* and *S. aureus*	/	[160]
CNC-based hydrogel	Tobramycin (TOB)	Cellulose nanocrystals (CNC)-based hydrogel	*S. aureus,* and *P. aeruginosa*	*P. aeruginosa*-infected wounds	[162]
QCS/OD/TOB/PPY@PDA hydrogel	TOB	Quaternized chitosan (QCS)/oxidized dextran (OD) hydrogel	*P. aeruginosa*, *S. aureus*, and *E. coli*	*P. aeruginosa*-infected burn wounds	[163]
MCGC hydrogel	Chlorogenic acid (CGA)	A CS matrix crosslinked with genipin (GP) hydrogel	*E. coli,* and *S. aureus*	*S. aureus*-infected diabetic wounds	[164]
E−4PBA nanofibers	E−4PBA	Poly(ε-caprolactone) (PCL) nanofibers	*S. aureus,* and MRSA	MRSA-infected wounds	[165]
RIF-CIP/RS100	CIP and rifampicin (RIF)	Eudragit® RS100-based fibers and Eudragit® RS100-based microparticles	*S. aureus,* and *E. coli*	Chronic infected diabetic wound	[166]
HA@Mup@SFM-AgNPs MNs	Mupirocin (Mup)	Hyaluronic acid (HA)-based MNs	*S. aureus,* and *E. coli*	*S. aureus*-infected wounds	[167]

### 3.1 Antibacterial agents loaded on inorganic NPs

#### 3.1.1 Antibacterial agents loaded on MOF

MOFs are an attractive type of nanocarrier for the delivery of antibacterial agents due to their large surface area, customizable pore sizes, and versatile properties. Huang et al. developed a nanoscale gallium-based MOF (GaMOF) as a novel carrier for gentamicin (Gen) to combat persistent bacterial infections, including intracellular and biofilm-associated bacteria [153]. The GaMOF NPs, with a high Brunauer–Emmett–Teller (BET) surface area of 1299.53 m^2^ g^−1^, were found to be effective in enhancing the permeability of antibiotics across cell membranes and biofilms, thereby increasing their bactericidal potency. The Ga^3+^ ions, with their ionic radii closely matching that of iron, can dupe certain bacterial absorption systems that cannot differentiate between the two elements, effectively serving as a covert "Trojan horse". Therefore, Ga^3+^ ions can interfere with bacterial iron uptake systems, thereby enhancing the efficacy of conventional antibiotics. In vivo and in vitro tests demonstrated that GaMOF, when combined with antibiotics, significantly improved bacterial clearance, alleviated inflammation, inhibited pyroptosis, and promoted wound healing. Similarly, Li et al. integrated benzalkonium chloride (BC) and MOF NPs (PCN-224) onto poly(ε-caprolactone) (PCL) electrospun nanofibrous membranes (ENMs) using a tannic acid-assisted adhesion strategy, termed PTM composites, to combat drug-resistant bacterial infections [154]. The PTM composites combined the photocatalytic activity of PCN-224 to generate singlet oxygen for PDT and the membrane-disrupting properties of the long hydrophobic alkyl chains on BC, which synergistically inhibited bacterial growth. In vitro and in vivo studies showed that PTM composites achieved bactericidal rates exceeding 99.99 % against *E. coli*, *S. aureus*, and MRSA within only 10 min of irradiation.

**Fig. 10:**
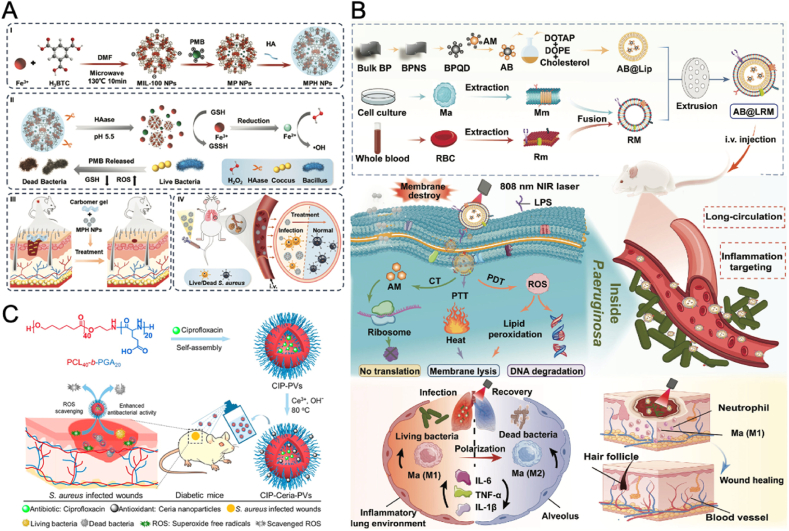
(A) Schematic illustration of the synthesis of MPH NPs and their therapeutic mechanisms in the infected wound healing and lung infection treatment [155]. Copyright 2024, Wiley-VCH. (B) Schematic illustration of the fabrication of AB@LRM NPs, their antibacterial mechanisms, and applications for bacterial pneumonia and infected-wound healing [158]. Copyright 2024, Wiley-VCH. (C) Schematic illustration of the synthesis of CIP-Ceria-PVs and their applications for infected diabetic wounds healing by combining the antioxidant-antibiotic treatment [159]. Copyright 2021, American Chemical Society.

Combining the intrinsic catalytic or photothermal activities to enhance antibacterial efficacy offers a targeted and effective treatment strategy for treating bacterial infections. For example, Guo et al. synthesized synergistic antibacterial NPs (MPH NPs) to combine antibiotic therapy with CDT for treating AMR bacterial infections (Fig. 10A) [155]. The MPH NPs were prepared by loading the antibacterial peptide polymyxin B (PMB) onto iron-based MOF (Fe-MOF, MIL-100) and modifying it with HA. The dual-function of CDT and antibiotic release allows the MPH NPs to exhibit strong antibacterial efficacy against *S. aureus* (98.5 %) and *E. coli* (100 %), as well as MRSA (98.4 %). In mice with infected wounds, the NPs significantly accelerated wound healing by enhancing collagen deposition, reducing inflammation, and promoting blood vessel formation. In another work, Ag_2_S@ZIF-Van that incorporated Ag_2_S quantum dots (QDs), vancomycin (Van), and ZIF-8 was synthesized for imaging and treating bacterial-induced inflammation and infections [156]. The Ag_2_S@ZIF-Van exhibited excellent second near-infrared (NIR-II) fluorescence around 1200 nm, along with high photothermal conversion efficiency and good biocompatibility. Subsequently, the nanosystem enabled enabled precise NIR-II fluorescence imaging of bacterial inflammation and showed synergistic antibacterial effects with Van, accelerating wound healing in infected models.

#### 3.1.2 Antibacterial agents loaded on other inorganic NPs

In addition to MOFs, other inorganic NPs can also be applied for integrating antibacterial agents for enhanced infected wounds management. For example, Deng et al. integrated azithromycin (AZM) into biomineralized iron sulfide NPs (FexSy) and GOx, named GOx@FexSy/AZM, for managing diabetic wounds [157]. In the early stages of infection, upon encountering glucose in the wound environment, GOx@FexSy/AZM can produce ROS, and release AZM and hydrogen sulfide (H_2_S) gas to dismantle biofilms, enhancing antibacterial efficacy by breaking down biofilms and killing bacteria like *S. aureus*. Moreover, H_2_S and the sustained release of AZM mitigated the hyperinflammatory response by inhibiting pro-inflammatory transcription factors in macrophages and promoting macrophage polarization toward the reparative M2 phenotype. In a mouse model with diabetic wound infections, GOx@FexSy/AZM effectively reduced bacterial load, suppressed inflammation, and accelerated wound healing in diabetic mice models.

Combining PTT or PDT therapy with antibiotics offers significant promise for combating bacterial infections. Liu et al. utilized a hybrid membrane composed of red blood cells (RBC) and macrophages (Ma) to encapsulate liposomes loaded with antibiotic amikacin (AM) and black phosphorus quantum dots (BPQDs) (AB@LRM) for the targeted treatment of *P. aeruginosa* infections (Fig. 10B) [158]. This hybrid membrane structure endowed the AB@LRM with immune escape abilities and bacterial targeting functions due to the long circulation capabilities of RBC membranes and the specific bacterial targeting of Ma membranes. Upon NIR irradiation, BPQDs generate ROS and heat, enhancing bacterial sensitivity to AM and facilitating bacterial membrane disruption, ensuring efficient bacterial eradication. In vitro studies demonstrated that AB@LRM significantly reduced the minimum inhibitory concentration of AM and achieved an antibacterial ratio of over 95 % against *P. aeruginosa*. In a mouse model with wound infections, AB@LRM-treated wounds showed significant bacterial reduction, decreased inflammation, enhanced angiogenesis, and thus a faster healing process compared to control groups.

Overall, inorganic NPs, with their high surface area, tunable properties, and inherent antibacterial activities, enable enhanced delivery and synergistic antibacterial effects through functionalities like ROS generation, gas release, and biofilm disruption. However, challenges such as controlled drug release, biocompatibility, and adaptability to diverse wound environments persist. Advancing these platforms requires a focus on integrating multifunctionality with clinical scalability and safety.

### 3.2 Antibacterial agents loaded in organic nanomaterials

The healing of infected diabetic wounds is particularly challenging due to high ROS levels and recurring bacterial infections. To address this challenge, Wang et al. designed ciprofloxacin (CIP)-loaded polymer vesicles decorated with ceria nanoparticles (CIP-Ceria-PVs) to combine antioxidant and antibiotic therapies (Fig. 10C) [159]. The polymer vesicles were constructed from amphiphilic poly(ε-caprolactone)-block-poly(glutamic acid) (PCL-b-PGA), creating a stable nanostructure with CIP encapsulated inside. Ceria NPs on the surface endowed the vesicles with high SOD-like activity, allowing them to scavenge superoxide free radicals. In vitro studies revealed that CIP-Ceria-PVs exhibited potent antibacterial activity against *E. coli* and *S. aureus*, with minimum inhibitory concentration (MIC) values lower than free CIP, due to the synergistic action of CIP and ceria. In diabetic mice with *S. aureus*-infected wounds, CIP-Ceria-PVs accelerated wound closure (complete healing in 14 days), improved collagen deposition and re-epithelialization, and effectively reduced superoxide levels, outperforming free CIP and control treatments. Fan et al. employed a unique hyperbranched dendritic-linear-dendritic copolymers (HBDLDs)-based hydrogels for controlled antibiotic delivery [160]. Hydrophilic novobiocin sodium salt (NB) and hydrophobic ciprofloxacin (CIP) were encapsulated in dendritic nanogels (DNGs) and incorporated into the hydrogel (DNGs-CIP). This combination enabled the rapid release of NB and sustained release of CIP, providing a strategic approach to treating bacterial infections at different stages of wound healing. The hydrogel's elasticity and swelling properties could be adjusted to mimic human skin, making it highly suitable as wound dressing. In direct comparison to commercial bandages, the DNGs-CIP hydrogel demonstrated markedly enhanced antibacterial activity, as evidenced by larger bacterial inhibition zones and more effective bacterial colony reduction in vitro. In addition, the DNGs-CIP promoted human dermal fibroblast (hDF) and keratinocyte (HaCaT) cell proliferation, underscoring its potential to accelerate tissue regeneration.

Organic nanomaterials offer flexibility for combining antibiotics with additional functionalities, such as controlled release and oxidative stress modulation, to address complex wound environments. However, challenges such as stability and scalability must be addressed to advance these systems into clinical practice. Tailored designs that optimize therapeutic outcomes while ensuring safety remain a key focus for future development.

### 3.3 Antibacterial agents loaded in 3D-structured nanocarriers

Furthermore, nanomaterials can be integrated into advanced wound care systems, such as hydrogels, microneedles, and electrospun nanofibrous dressings, providing a multifaceted approach that combines antimicrobial activity with enhanced wound healing capabilities [[49], [50], [51], [52], [53], [54], [55], [56]]. Besides, wound dressings that utilize nanomaterials can provide prolonged antimicrobial effects, maintain moisture, and enhance cell proliferation and the formation of new blood vessels, thereby speeding up the healing process of wounds.

#### 3.3.1 Antibacterial agents loaded in hydrogels

Hydrogels, created from either natural or synthetic polymers, represent an exciting class of materials that are particularly well-suited for delivery of antibacterial agents due to their exceptional ability to create self-sustaining, 3D viscoelastic frameworks [55,161]. For example, Li et al. proposed a sprayable antibacterial hydrogel formulated by simply mixing cellulose nanocrystals (CNC) with aminoglycoside antibiotics, such as tobramycin (TOB), for treating infected wounds [162]. The hydrogel was synthesized through simple ionic interactions between the sulfate groups on CNCs and the amine groups on aminoglycosides, which created a robust and biocompatible structure with desirable shear-thinning and self-healing properties, enabling easy administration by injection or spraying. The CNC-based hydrogel exhibited sustained TOB release for up to four days, maintaining antibacterial efficacy against *S. aureus* and *P. aeruginosa*. In subcutaneous infection models and *P. aeruginosa*-infected wound models, the hydrogel accelerated wound healing, reduced inflammation (lower IL-1β, TNF-α, and IL-6 levels), and improved tissue regeneration compared to free TOB or CNC suspensions.

**Fig. 11:**
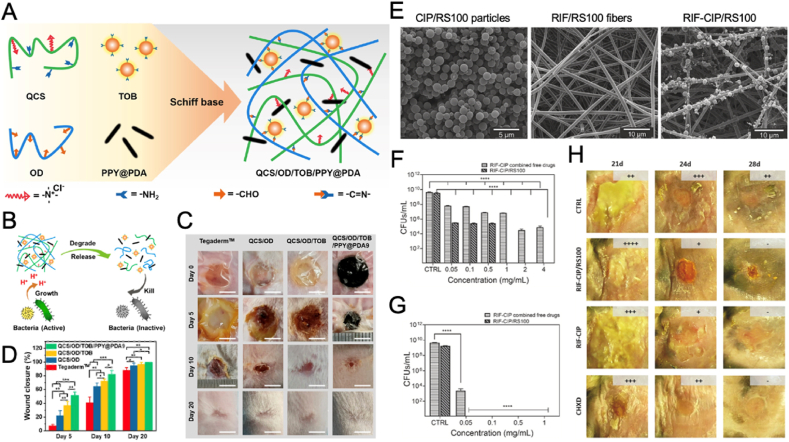
(A) Schematic illustration of the fabrication of QCS/OD/TOB/PPY@PDA hydrogel. (B) Antibacterial mechanism of QCS/OD/TOB/PPY@PDA hydrogel. (C) Photographs of wounds in mice receiving different treatments. Scale bar: 5 mm. (D) Wound contraction of mice receiving different treatments [163]. Copyright 2022, American Chemical Society. (E) SEM images of CIP/RS100 particles, RIF/RS100 fibers, and RIF-CIP/RS100. Biofilm formation inhibition effects in (F) *S. aureus* and (G) *E. coli*. (H) Photographs of the wounds at different time points in chronic infected murine models receiving different treatments, including CTRL, RIF-CIP/RS100, free antibiotics (RIF-CIP), and chlorhexidine (CHXD) [166]. Microbiological results were shown as insets. (−) no growth; (+) mild bacterial growth; (++) moderate bacterial growth; (+++) massive bacterial growth; (++++) extensive bacterial growth. Copyright 2023, Elsevier.

Similarly, a self-healing hydrogel (QCS/OD/TOB/PPY@PDA hydrogel) based on quaternized chitosan (QCS), oxidized dextran (OD), TOB, and polydopamine-coated polypyrrole nanowires (PPY@PDA NWs) were synthesized for treating deep cutaneous fungal infections and infected burn wounds (Fig. 11A) [163]. TOB was incorporated into the hydrogel through Schiff base cross-linking, allowing for a controlled release in the acidic microenvironment (Fig. 11B). This on-demand release system ensures that TOB is only released in the presence of active bacterial infections, minimizing the risk of antibiotic overuse and reducing the potential for antibiotic resistance. In addition, the NWs provided antioxidant and photothermal properties, enabling antibacterial, anti-inflammatory, and tissue regeneration effects. Moreover, the hydrogel sustained its antibacterial properties for up to 11 days and could be activated by NIR irradiation to further enhance its bactericidal effects. In a *P. aeruginosa*-infected burn wound mouse model, the hydrogel outperformed standard dressings like Tegaderm, significantly reducing inflammation and accelerating wound healing (Fig. 11C and D).

Combining the catalytic capabilities of MOFs and the antibacterial properties of antibacterial agents provides a promising solution for managing infected diabetic wounds. Wei et al. integrated the Mn-N4 metalloporphyrin MOFs and chlorogenic acid (CGA) in a CS matrix crosslinked with genipin (GP) hydrogel to fabricate a dual-metal MOF-nanozyme-decorated hydrogel (MCGC), which demonstrated a high H_2_O_2_ scavenging rate, converting excess ROS into oxygen to promote cellular viability and reduce inflammation [164]. The integrated CGA provided effective antibacterial action, particularly against *E. coli* and *S. aureus*, with bacterial inhibition rates over 99 %. The addition of CGA contributed potent antibacterial effects, particularly against *E. coli* and *S. aureus*, with bacterial inhibition rates exceeding 99 %. More importantly, MCGC treatment significantly lowered pro-inflammatory cytokines (IL-1β, IL-6, TNF-α), highlighting the hydrogel's capacity to modulate the immune response.

#### 3.3.2 Antibacterial agents loaded in nanofibers

Electrospun nanofibers, with their high surface area-to-volume ratio, significant porosity, and 3D scaffold-like structure that mimics natural tissue, hold great potential as an innovative approach for treating acute and chronic wounds [51]. Recent developments highlight their effectiveness as scaffolds for delivering antibiotics, enhancing antibacterial efficacy and promoting wound repair. Dong et al. incorporated a multi-armed antibiotics (E−4PBA) into poly(ε-caprolactone) (PCL) nanofibers to create an in situ nanofibrous dressing for combating multidrug-resistant bacterial infections [165]. The nanofibrous dressing can be directly applied to various wound shapes and sizes using a handheld electrospinning device, offering adaptability in wound care and achieving a controlled release of the antibiotic for sustained protection. Compared to conventional gauze dressings, the nanofibrous dressing demonstrated superior antibacterial performance against *S. aureus* and MRSA, achieving higher bacterial inhibition rates. In infected wound models, it not only exhibited faster healing rates but also significantly reduced inflammation and enhanced collagen deposition and tissue regeneration compared to commercial gauze.

To enhance antibiotic delivery for chronic wound treatment, Miranda-Calderon et al. developed an electrospun wound dressing that incorporated a dual antibiotic release system [166]. This wound dressing involved encapsulating rifampicin (RIF) within Eudragit® RS100-based fibers (RIF/RS100 fibers) and CIP within Eudragit® RS100-based microparticles (CIP/RS100 particles), which were assembled to form the composite dressing, RIF-CIP/RS100 (Fig. 11E). This design enabled an initial burst release of CIP followed by a sustained release of RIF, providing a synergistic antimicrobial effect against *S. aureus* and *E. coli*, preventing biofilm formation, and minimizing the risk of recurring infections (Fig. 11F and G). Cytotoxicity tests confirmed the dressing's safety for human skin cells, including keratinocytes and fibroblasts, at antimicrobial doses. Mice with chronic infected wound treated with the dressing exhibited faster wound healing, with reduced bacterial load and enhanced tissue regeneration compared to groups treated with free antibiotics or conventional antiseptics like chlorhexidine (Fig. 11H).

#### 3.3.3 Antibacterial agents loaded in microneedles

Loading antibacterial agents in MN systems can achieve highly efficient drug delivery to damaged wounds. Li et al. developed a novel dissolving MN system that integrates silk fibroin microspheres (SFM) decorated with AgNPs and mupirocin (HA@Mup@SFM-AgNPs MNs) for the synergistic treatment of bacterial biofilm infections [167]. The SFM serves as a biological template for AgNPs synthesis, which provided broad-spectrum antibacterial effects by promoting bacterial adhesion and subsequent eradication. The MN system, made of HA and containing SFM-AgNPs along with the antibiotic mupirocin, effectively penetrated and delivered antibacterial agents within the biofilm, demonstrating significant efficacy against *S. aureus*-infected wounds.

Hydrogels, nanofibers, and microneedles, are advanced platforms for antibacterial delivery and wound healing. Hydrogels enable sustained drug release and support tissue regeneration, nanofibers offer precise antibiotic delivery and biofilm prevention, and microneedles target biofilm-associated infections effectively. While promising, these systems require further refinement in scalability and adaptability to diverse wound types to realize their full clinical potential.

## 4 Conclusion and perspective

Nanomaterials are emerging as effective tools for managing bacterial infections during wound healing, providing unique mechanisms of action and multifunctionality that address the limitations of conventional antibiotics [19,22,23,27,28]. The versatility of nanomaterials allows for diverse strategies, including the ion release, ROS generation, PTT and PDT, disruption of membrane, and targeted drug delivery. These approaches not only provide effective antibacterial effects but also help overcome issues related to biofilm formation and antibiotic resistance, both of which are major obstacles in infected-wound management. Studies discussed in this review have demonstrated that nanomaterials, such as inorganic NPs, organic NPs, hybrid nanomaterials, and various nanocarriers, in accelerating the healing of bacterial-infected wounds. The incorporation of these nanomaterials into wound care products not only led to enhanced antibacterial efficacy, but also achieved improved tissue regeneration, and accelerated wound closure. Despite these advancements, several challenges remain in the development of nanomaterials for wound care.

### 4.1 Challenges for nanomaterials in infected wounds management

#### 4.1.1 Combatting MDR pathogens

The rise of MDR pathogens, including MRSA, vancomycin-resistant *Enterococci* (VRE), multidrug-resistant *Acinetobacter baumannii* (MDR-AB), multidrug-resistant *P*. *aeruginosa* (MDR-PA) and carbapenem-resistant *Enterobacteriaceae* (CRE), pose a significant challenge to the efficacy of traditional antibiotics in managing infected wounds [6,23,168,169]. Nanomaterials offer innovative solutions due to their multifaceted mechanisms that are less prone to resistance development. For instance, AgNPs exhibit exceptional antibacterial activity against MRSA by releasing Ag^+^ ions [60,61,63,138]; nanomaterials designed for CDT [75,77,81], PDT [91,95], PTT [97,104,114,117], electrostatic interactions-mediated therapy [45], and combination therapy [40,47,140,142], effectively eradicate MRSA by generating ROS, producing localized heat, or destabilizing bacterial membranes; synergistic platforms that combine nanomaterials with existing antibiotics further enhance therapeutic efficacy against MRSA [154,155,165]. While promising, several challenges persist. Excessive ROS production, while effective against bacteria, risks collateral damage to host tissues, particularly in chronic wounds with prolonged exposure. The heterogeneity of biofilms associated with MDR pathogens complicates effective nanomaterial design, as biofilm matrices vary in composition and density under different conditions, limiting penetration and therapeutic efficacy. Although substantial progress has been made against MRSA, limited research addresses other MDR pathogens such as VRE, MDR-AB, and MDR-PA. Future research should prioritize tailoring nanomaterials to diverse bacterial species, broadening the scope beyond MRSA, and integrating these systems into clinical practice through scalable and biocompatible designs.

#### 4.1.2 Overcoming biofilm barriers

Bacterial biofilms present a significant challenge in managing infections due to their complex EPS matrix, which acts as a physical and biochemical barrier, shielding bacteria from antibiotics and the immune system [[9], [10], [11], [12], [13]]. Nanotechnology offers promising solutions to overcome these barriers by leveraging innovative strategies [170]. For instance, nanoplatforms with EPS matrix degradation property, such as those utilizing enzymes [119] and microorganism LG [143], have demonstrated the ability to degrade EPS components, enhancing drug penetration and bacterial eradication. Stimuli-responsive nanomaterials activated by internal triggers, such as pH [63], and glucose [157], or external triggers like light [139,147,148,151] and ultrasound [42] precisely release therapeutic agents or biofilm-disrupting molecules at the infection site, improving targeting and minimizing off-target effects. Surface-modified NPs, especially positively charged ones, exploit electrostatic interactions to destabilize the biofilm matrix and enhance drug retention [45]. Emerging technologies like microneedles provide active delivery systems capable of physically penetrating dense biofilm matrices and delivering therapeutic payloads directly to the infection site [167]. Evidences from in vitro and in vivo studies has demonstrated that these approaches can significantly reduce biofilm biomass and bacterial load. Multifunctional platforms that integrate these strategies hold great promise for addressing the unique challenges posed by biofilms and improving clinical outcomes in wound infection management.

#### 4.1.3 Environmental factors influencing nanomaterial performance

Environmental factors such as pH and temperature play critical roles in determining the performance of nanomaterials in wound healing applications. For example, acidic microenvironments in infected wounds often enhance the activity of pH-responsive nanomaterials, such as ion-releasing systems or ROS-generating therapies [63,76,77,81,140,163]. However, in chronic wounds with an alkaline microenvironment, the therapeutic performance of these materials may decrease, necessitating designs that adapt to fluctuating pH conditions [76,77,81,140]. Variations in environmental temperature can significantly influence the stability and activity of nanomaterials. For instance, high temperatures may accelerate the release of heat-sensitive nanocarriers leading to accelerated release of therapeutic components [96,97]. Designing nanomaterials that are stable and responsive under variable pH and temperature conditions is crucial for maintaining their therapeutic efficacy and ensuring consistent wound management outcomes.

#### 4.1.4 Optimizing physicochemical properties

The physicochemical properties of nanomaterials, including size, shape, surface charge, and advanced features like optical, thermal, and electrical characteristics, significantly influence their antibacterial efficacy. Smaller NPs enhance biofilm penetration and bacterial membrane interaction due to their high surface area-to-volume ratios, while anisotropic shapes, such as nanorods disrupt membranes more effectively with their sharp edges and aspect ratios [60,63,171]. Positively charged nanomaterials bind strongly to negatively charged bacterial membranes, causing structural destabilization and cell death [[43], [44], [45], [46], [47]]. Optical properties are crucial for PTT and PDT, enabling ROS generation or heat production under light stimuli. Electrical properties, such as piezoelectricity, further enhance bacterial disruption through localized ROS production under ultrasound [42,[123], [124], [125]]. Optimizing these properties attributes enhances specificity, reduces off-target effects, and improves biocompatibility of tailored nanomaterials for specific wound infections.

#### 4.1.5 Integrating antibacterial and regenerative functionalities

Effective management of infected wounds requires a dual focus on antibacterial efficacy and tissue repair. Balancing these priorities is particularly challenging, as mechanisms like ROS generation, while effective against bacteria, can damage host tissues, delay healing, and impair critical cellular processes such as fibroblast proliferation and angiogenesis. Addressing this challenge requires innovative solutions, such as dynamic wound-responsive nanomaterials that adapt to stimuli like pH or ROS for targeted and sustained effects. Incorporating bioactive ions (such as Cu^2+^) or growth factors can enhance angiogenesis and collagen synthesis while retaining antibacterial properties [43,91,97,114,144,160]. Additionally, the use of biocompatible and degradable materials in nanomaterial design can further ensure that the dual functionalities of antibacterial and regenerative properties are achieved without compromising host tissue health or repair processes.

#### 4.1.6 Ensuring safety, stability, and scalability

Safety, stability, and long-term effects remain critical concerns for clinical translation of nanomaterials. Although ROS produced by nanomaterials are highly effective in damaging bacterial cells, excessive ROS can also damage host tissues, leading to delayed wound healing or chronic inflammation. Moreover, systemic application of nanomaterials raises concerns about their long-term impact on vital organs such as the liver, kidneys, and lungs. Additionally, prolonged exposure to certain NPs may result in cytotoxicity or immune dysregulation, particularly in chronic wound environments, as the degradation and clearance mechanisms of nanomaterials remain poorly understood, raising concerns about their persistence in the body and potential long-term risks. Dynamic physiological conditions, such as pH fluctuations, enzymatic activity, and temperature variations, can influence the stability of nanomaterials and alter their therapeutic efficacy. To address these challenges, strategies such as surface modifications with biocompatible coatings, the use of biodegradable polymers, and the development of stimuli-responsive or controlled-release nanoplatforms can minimize off-target effects and improve therapeutic outcomes. Comprehensive toxicological assessments and long-term studies are necessary to evaluate cytotoxicity, oxidative stress, and inflammatory responses, ensuring the safety and efficacy of nanomaterials in wound care applications. Furthermore, establishing standardized protocols for evaluating nanomaterial stability, bioaccumulation, and clearance is crucial to facilitate clinical adoption.

#### 4.1.7 Tailoring design for specific infections

Infected wounds vary significantly depending on the causative microorganisms and underlying conditions, such as diabetes, requiring tailored therapeutic strategies [8]. For example, Gram-positive bacteria, with their thick peptidoglycan layers, may require nanomaterials capable of enzymatic degradation or specific ion release profiles to disrupt their cell walls; conversely, Gram-negative bacteria possess an outer membrane containing lipopolysaccharides, which necessitates nanomaterials designed to penetrate this additional barrier while minimizing host cell toxicity [172,173]. Additionally, the EPS matrix varies between bacterial species and affects nanomaterial penetration, retention, and therapeutic efficacy. Strategies such as surface modifications to target specific bacteria or incorporating enzymes capable of degrading the biofilm matrix can significantly enhance treatment outcomes. Furthermore, designing stimuli-responsive nanomaterials that activate in response to localized microenvironmental cues, such as pH and ROS, can enable precise targeting of biofilms without off-target effects.

#### 4.1.8 Synergistic effects in multifunctional nanomaterials

The combination of multiple antibacterial mechanisms within a single nanoplatform offers a promising strategy to enhance therapeutic outcomes in managing wound infections, especially those associated with biofilms and MDR bacteria. For example, nanomaterials integrating PTT with CDT or PDT achieve simultaneous biofilm disruption and bacterial eradication by generating localized heat and ROS under NIR light activation [40,140,142]. Beyond antibacterial effects, synergistic nanomaterials can promote wound healing through the controlled release of bioactive ions or the incorporation of immune-modulating agents, fostering a favorable microenvironment for tissue regeneration [43,91,97,114,144,160]. However, the integration of multiple components may increase material complexity, potentially affecting stability, scalability, and biocompatibility. Furthermore, precise control over the activation and interplay of these mechanisms is critical to avoid off-target effects, such as excessive ROS production that could damage host tissues. Designing multifunctional nanoplatforms that are not only effective and safe but also easy to synthesize is critical for future research.

#### 4.1.9 Techniques to assess antibacterial activity

Establishing standardized evaluation protocols is critical for enabling consistent, reliable comparisons across studies and facilitating the clinical translation of nanomaterial-based therapies for infected wound management. The antibacterial efficacy of nanomaterials is commonly assessed through standard microbiological methods such as disk diffusion, broth microdilution, and colony-forming unit (CFU) counting to quantify bacterial susceptibility. Biofilm disruption studies employ imaging techniques like confocal laser scanning microscopy (CLSM) and SEM, along with assays like crystal violet staining, to evaluate biofilm penetration and degradation. Mechanistic insights are gained through ROS detection and live/dead bacterial staining, while in vivo models of infected wounds, such as murine or rat models, are used to validate efficacy and biocompatibility in complex biological environments. Moreover, to fully assess the therapeutic potential of nanomaterials, comparative studies against conventional wound therapies, such as antibiotics and standard wound dressings, are essential. These evaluation protocols provide a clear understanding of the advantages, limitations, and potential translational value of nanomaterials in addressing infection-related challenges.

#### 4.1.10 Influence of application methods

The method of nanomaterial application plays a crucial role in the therapeutic outcomes of infected wounds. Topical applications, such as hydrogels, nanofibers, and sprays, are highly effective for localized treatment, allowing sustained and controlled release of therapeutic agents directly at the wound site while minimizing systemic toxicity [174], which are particularly beneficial for treating surface infected wounds. Injectable nanomaterials, on the other hand, offer superior systemic distribution and are better suited for treating subcutaneous or systemic infections [175]. However, injectable approaches may face challenges in achieving targeted delivery to wound site and penetrating dense biofilms without affecting surrounding healthy tissue. Tailoring nanomaterial application strategies to the specific characteristics of the wound environment is therefore essential to optimize therapeutic outcomes, ensuring effective delivery while minimizing potential side effects.

#### 4.1.11 Economic and clinical considerations

Economic factors are crucial for the clinical transformation of nanomaterials for wound care. While nanomaterials provide unique advantages in the management of infected wounds, their production often involves high costs due to the complexity of synthesis and functionalization. Compared to conventional treatments, such as antibiotics and standard wound dressings, these advanced platforms may initially appear less cost-effective. However, nanomaterials could offer long-term savings by accelerating wound healing, reducing complications, and addressing infections caused by MDR pathogens, thereby shortening treatment duration and hospital stays. Integrating multifunctional properties into single platforms may also simplify treatment, improving cost efficiency. Efforts to reduce production costs, such as scalable manufacturing and green synthesis, are ongoing. Collaboration between researchers, manufacturers, and healthcare providers is essential to balance efficacy, safety, and affordability, ensuring these advanced therapies become practical for widespread use.

### 4.2 Future research directions

Although limited examples currently available in clinical use or trials, the clinical translation of nanomaterials for wound infections management has seen promising developments [176]. Acticoat^TM^, a commercially available nanomaterial-based wound care product, incorporates AgNPs and has been approved for the treatment of burns and microbial-infected wounds due to its potent antimicrobial properties [177], demonstrating the potential for nanomaterials to address infections in real-world settings by leveraging their unique antibacterial mechanisms. Beyond wound healing, other nanomaterial-based products highlight the broader applicability of nanotechnology in infection management [176]. For instance, Sebacia Microparticles, composed of gold-coated silica NPs, have been approved for the topical PTT of acne vulgaris [178], showcasing the feasibility of PTT-based strategies for localized infections. Additionally, liposomal amikacin [179] and liposomal amphotericin B [180] have been widely applied for treating fungal infections, setting benchmarks for the clinical deployment of nanomedicines. While these examples illustrate the translational potential of nanomaterials, efforts to optimize their safety, scalability, and therapeutic efficacy remain essential to expand their applications specifically for wound healing. By addressing these challenges, the clinical adoption of nanotechnology-based solutions for managing infected wounds can be further accelerated.

Future research in nanomaterials for infected wound management should prioritize the development of multifunctional platforms that effectively address key challenges, including MDR pathogens, biofilm barriers, and tissue regeneration. Advanced systems leveraging stimuli-responsive mechanisms, such as pH, ROS, or enzymatic activity, can enable precise, targeted therapies while minimizing off-target effects. Examples like Acticoa^TM^ and Sebacia Microparticles highlight the translational potential of nanotechnology in wound care, demonstrating its ability to bridge the gap between laboratory research and clinical application. High-potential nanomaterials, such as AgNPs for their broad-spectrum antibacterial activity and Au-based platforms for PTT, offer significant promise for clinical use. However, challenges related to scalability, affordability, and long-term safety must be addressed. Strategies such as green synthesis, streamlined production processes, and rigorous preclinical evaluations are essential to overcome these barriers. By tailoring nanomaterials to specific wound environments and integrating them with existing treatment modalities, nanomedicine has the potential to transform the management of infected wounds and improve clinical outcomes.

### 4.3 Conclusion

In conclusion, nanomaterials represent a significant advancement in the field of wound care, offering promising solutions for bacterial-infected wounds that are resistant to traditional treatments. With their unique physicochemical properties and multifunctional capabilities, nanomaterials offer new avenues for tackling infections and promoting tissue repair. With continued research and collaboration, nanomaterials have the potential to significantly improve infected wounds management and provide alternative approaches for treating complex and chronic wounds.

## CRediT authorship contribution statement

**Jianping Zhu:** Writing – review & editing, Writing – original draft, Investigation, Funding acquisition. **Fan Xia:** Writing – review & editing, Writing – original draft, Visualization, Investigation, Conceptualization. **Shuaifei Wang:** Writing – review & editing, Investigation, Funding acquisition. **Yan Guan:** Writing – review & editing, Conceptualization. **Fuqiang Hu:** Writing – review & editing, Conceptualization. **Fangying Yu:** Writing – review & editing, Writing – original draft, Investigation, Conceptualization.

## Declaration of competing interest

The authors declare that they have no known competing financial interests or personal relationships that could have appeared to influence the work reported in this paper.

## Data Availability

Data will be made available on request.
